# Concentration-dependent transcriptional switching through a collective action of cis-elements

**DOI:** 10.1126/sciadv.abo6157

**Published:** 2022-08-10

**Authors:** Kevin Rodriguez, Albert Do, Betul Senay-Aras, Mariano Perales, Mark Alber, Weitao Chen, G. Venugopala Reddy

**Affiliations:** ^1^ Department of Botany and Plant Sciences, University of California Riverside, Riverside, CA 92521, USA.; ^2^ Department of Mathematics, University of California Riverside, Riverside, CA 92521, USA.; ^3^ Interdisciplinary Center for Quantitative Modeling in Biology, University of California Riverside, Riverside, CA 92521, USA.

## Abstract

Gene expression specificity of homeobox transcription factors has remained paradoxical. WUSCHEL activates and represses *CLAVATA3* transcription at lower and higher concentrations, respectively. We use computational modeling and experimental analysis to investigate the properties of the cis-regulatory module. We find that intrinsically each cis-element can only activate *CLAVATA3* at a higher WUSCHEL concentration. However, together, they repress *CLAVATA3* at higher WUSCHEL and activate only at lower WUSCHEL, showing that the concentration-dependent interactions among cis-elements regulate both activation and repression. Biochemical experiments show that two adjacent functional cis-elements bind WUSCHEL with higher affinity and dimerize at relatively lower levels. Moreover, increasing the distance between cis-elements prolongs WUSCHEL monomer binding window, resulting in higher *CLAVATA3* activation. Our work showing a constellation of optimally spaced cis-elements of defined affinities determining activation and repression thresholds in regulating *CLAVATA3* transcription provides a previously unknown mechanism of cofactor-independent regulation of transcription factor binding in mediating gene expression specificity.

## Teaser

Optimally spaced cis-elements of defined affinities regulate concentration-dependent transcriptional switching in stem cells.

## INTRODUCTION

Spatiotemporal regulation of gene expression is critical for specifying different cell types during development (*1*–*3*). Eukaryotic gene regulation involves interactions among DNA sequences and proteins, many of which are transcription factors (TFs). Enhancers, the DNA sequences that bind a given TF or multiple TFs, can regulate transcription irrespective of their location in the gene (*1*, *3*, *4*). Since a given class of TFs binds similar DNA sequences, how they achieve gene expression specificity has been the subject of intense investigation. One of the possible mechanisms to achieve specificity is the binding of cofactors that may unmask latent binding specificity of TFs as shown in the case of homeobox-mediated regulation in anterior-posterior body patterning in *Drosophila melanogaster* (*5*). Another mechanism involves the utilization of the cis-regulatory modules (CRMs), a subset of enhancers that contain cis-elements for one or more TFs, which have been shown to determine the expression of neighboring genes in a variety of organisms (*4*, *6*–*9*). In general, the CRMs can be classified into homotypic, where they bind a given type of TF, or heterotypic, where they bind different TFs (*10*, *11*). The heterotypic CRMs largely have been thought to mediate spatiotemporal regulation of gene expression through their ability to recruit different collections of TFs in space and time (*10*, *12*, *13*).

Both the homotypic and heterotypic CRMs have been shown to regulate spatiotemporal gene expression patterns in response to TF gradients. The earliest examples of homotypic CRMs have been described in the promoters of genes activated by the TFs that accumulate in a graded manner during early embryonic development in *Drosophila* (*14*–*17*). Classically, the French flag model proposed by Wolpert has been applied to explain the expression of genes by TF gradients. According to this model, the target gene expression is highest in places of the highest concentration of the TF (*18*). Analysis of multiple CRMs has identified three recurring properties: cis-element number, affinity, and cooperativity, which determine gene expression (*16*). Essentially, decreasing any of the three CRM properties reduces the mean expression while increasing any of the properties leads to overexpression (*15*, *16*, *19*, *20*).

**Fig. 1.**
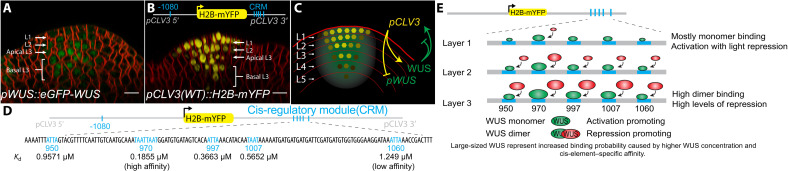
CRM required for *CLV3* activation and repression. Side views of wild-type (WT) meristems with the WUS protein reporter *pWUS::eGFP-WUS* (**A**) and CLV3 transcriptional reporter containing all five WT cis-elements within the 3′ CRM *pCLV3(WT)::H2B-mYFP* (**B**). Scale bars, 10 μm. (**C**) Side view of a SAM cartoon showing WUS protein distribution (green) and CLV3 (yellow), which form a regulatory feedback loop across cell layers. The *CLV3* CRM, a cluster of WUS binding cis-elements, interacts with the WUS concentration to repress and activate *CLV3*. CLV3 signals to WUS at both the posttranslational level, enriching the WUS protein, and the transcriptional level, repressing *WUS* expression. (**D**) Schematic of the *CLV3* gene including the location and *K*_d_ of WUS binding TAAT cis-elements (cyan) of the *CLV3* CRM. (**E**) Schematic of WUS monomer and dimer binding to the *CLV3* cis-elements depending on the WUS concentration gradient (across SAM cell layers) and the relative affinities of cis-elements.

In *Arabidopsis* shoot apical meristems (SAMs), WUSCHEL (WUS) is a homeodomain TF expressed in the rib meristem (RM) (*21*, *22*). WUS protein migrates into the overlying central zone (CZ), where it promotes stem cell fate by repressing differentiation and also activates its own negative regulator—*CLAVATA3* (*CLV3*) (*23*, *24*) (Fig. 1, A to C). CLV3 encodes a secreted peptide that activates a receptor kinase pathway to restrict *WUS* expression (*25*, *26*). WUS has also been shown to bind to the promoters of key differentiation-promoting TFs to repress transcription (*27*). How the same TF activates some genes, such as *CLV3*, and represses other genes in the same cells is largely unknown. However, a recent study has provided some clues to this regulation. Perales *et al*. (*24*) showed that WUS binds a CRM, a collection of five closely spaced cis-elements, in the *CLV3* enhancer region (Fig. 1D). The incremental deletion of cis-elements led to down-regulation of *CLV3* in the outer layers of the CZ and misexpression in the inner layers of the RM, suggesting that same cis-elements mediate activation and repression of *CLV3* at lower and higher WUS, respectively. Biochemical analysis revealed that WUS binds cis-elements as monomers at lower WUS concentrations and binds as dimers/multimers with increasing WUS concentrations, suggesting that dimerization/multimerization of WUS at higher levels may repress *CLV3* (Fig. 1E). The biochemical analysis also revealed that DNA promotes homodimerization (*28*, *29*). Furthermore, increasing the affinity of one of the cis-elements decreased the dimerization threshold and led to the repression of *CLV3* in the CZ, supporting the hypothesis of affinity-based concentration-dependent activation-repression of transcription in maintaining *CLV3* expression over a window of WUS levels. This concentration-dependent switching of *CLV3* transcription is unique among the homotypic CRMs studied and forms an exception to the French flag model.

Understanding how the concentration-dependent transcriptional switch is established requires understanding how the *CLV3* CRM functions as a unit. The complexity of the *CLV3* CRM regulation involving five cis-elements and the bidirectional relationship between *CLV3* and WUS can be challenging to untangle experimentally. The current experimental limitations cannot provide a direct real-time view of the actual WUS/*CLV3* molecular dynamics under precisely defined conditions. A multiscale computational model capable of simulating the binding and unbinding dynamics of WUS to all five cis-elements and *CLV3* transcription at the tissue level can be helpful in providing mechanistic insights into the WUS concentration–dependent functioning of the *CLV3* CRM.

Different approaches have been developed for studying TF binding dynamics. The thermodynamic models are usually based on the occupancy of the promoter by the TFs, the statistical weights of possible configurations, and the free energy (*30*–*34*). However, when multiple cis-elements with different affinities and their interactions are involved as observed in *CLV3* CRM, the number of possible configurations becomes large and it is not practical to use the thermodynamic approach. Instead, the stochastic simulation algorithm, i.e., Gillespie algorithm where the dynamics of WUS binding and unbinding to the cis-elements can be modeled as a series of probabilistic events occurring at random time steps determined, is ideal to explicitly model *CLV3* transcription.

We developed a stochastic model to simulate the WUS binding to the CRM in a single cell. The single-cell model was applied to simulate WUS binding cis-elements under different concentrations and compared the simulation output with the experimental data on tissue-level expression patterns of different cis-element mutants of *CLV3* to investigate the roles of WUS binding affinity, distance-dependent cooperativity among cis-elements, and RNA polymerase II (Pol II) recruitment in the transcription process. Subsequently the single-cell stochastic model was applied to multiple cells represented by unit spheres to develop a cell-based three-dimensional (3D) model representing the SAM. The 3D model was applied to further test the mechanisms identified in the single-cell model in generating the spatial patterns of *CLV3* expression.

Using a WUS gradient consistent with the experimental data, both computational models suggested a role for residence time limit (see Results for details) of WUS monomer binding to the individual cis-elements of different affinities, which have been shown to activate *CLV3* largely to a similar extent when acting alone. Beyond residence time limit, the aged WUS monomers fail to activate transcription and they are replaced with newly synthesized WUS monomers to sustain *CLV3* activation. Our experimental observations showing a correlation between higher WUS turnover and increased *CLV3* activation support such a mechanism. When multiple cis-elements are involved, we found that the cooperative binding of WUS monomers and dimers is required to achieve correct *CLV3* activation patterns. The model simulations also suggested a nonhomogeneous cooperativity among cis-elements that depends on the intervening distance between cis-elements. The model prediction on distance-dependent cooperativity was tested in experiments by increasing the intervening distance between cis-elements, which revealed an increase in *CLV3* activation. The corresponding biochemical experiments revealed that an increase in intervening distance between cis-elements increased their affinity to WUS monomers but did not alter the concentration at which WUS monomers switch to form stable dimers/higher molecular weight complexes. These results show the importance of optimal spacing between cis-elements in determining the concentration range over which an appropriate number of WUS monomers and dimers populate on cis-elements in setting up the activation-repression thresholds. The 3D model that incorporates multiple cis-elements of different affinities that are spaced optimally allowed independent manipulation of the monomer and dimer cooperativity. Our simulations revealed that monomer cooperativity was critical for expression of *CLV3* at the lower WUS concentration, while the dimer cooperativity was critical for repression at the higher WUS concentration. Moreover, a balance of the monomer and dimer cooperativity levels was critical to achieve the wild-type *CLV3* expression at a WUS concentration range observed in experiments.

## RESULTS

### Affinity and collective activity of multiple cis-elements determine *CLV3* expression

**Fig. 2.**
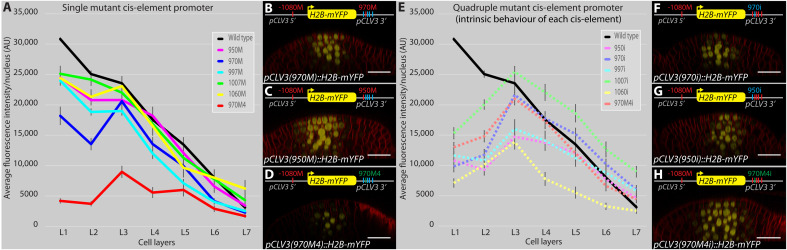
The number of cis-elements and affinity influences the collective behavior of the CRM in regulating *CLV3* activation and repression. Average fluorescence levels (mean ± SE) of H2B-mYFP in different cell layers of various *pCLV3::H2b-mYFP* promoter variants carrying a mutation in single cis-elements of the 3′ CRM (**A**). AU, arbitrary unit. (**B** to **D**) Side views of WT meristems showing various mutant *pCLV3::H2B-mYFP* reporter expression patterns. Single cis-element mutants 970M (B) and 950M (C) and a higher-affinity mutant 970M4 (D). Average fluorescence levels (mean ± SE) of H2B-mYFP in different cell layers of various *pCLV3::H2b-mYFP* promoter variants carrying mutations in four of the five cis-element mutants (quadruple mutants) (**E**). Side views of WT meristems showing various mutant *pCLV3::H2B-mYFP* reporter expression patterns. Quadruple mutant (mutants 950M, 997M, 1007M, and 1060M) referred to as 970 intrinsic (970i) (**F**), (mutants 970M, 997M, 1007M, and 1060M) referred to as 950 intrinsic (950i) (**G**), and (mutants 950M, 997M, 1007M, and 1060M) referred to as 970M4 intrinsic (970M4i) (**H**). All cis-element mutations within the CRM in the 3′ region were generated in the mutant-1080 cis-element background. In all images, scale bars = 20 μm. (A and E) The error bars represent the SE (in all cases, *n* = 4 represents independent transformants).

Incremental mutations of cis-elements within the CRM result in incremental down-regulation of *CLV3* expression in outer cell layers of the CZ and up-regulation in the inner cell layers of the RM, suggesting interaction among cis-elements (*24*). To understand the collective behavior of cis-elements, we first deduced the contribution of each cis-element within the CRM to the regulation of *CLV3* by analyzing the loss of binding mutations in each of the five cis-elements. Single loss of binding mutations in high-affinity cis-element 970 (Fig. 1D and table S1) led to drastic down-regulation in the outer layers of CZ (Fig. 2, A and B, and fig. S1). On the other hand, independent loss of binding mutations in the four lower-affinity cis-elements led to a minor down-regulation in the L1 layer (Fig. 2, A and C). These results show that all five elements contribute to the *CLV3* expression, with the highest-affinity cis-element contributing maximally over the lower-affinity cis-elements *CLV3*. Moreover, our previous work shows that increasing the affinity of 970 cis-element alone down-regulated *CLV3* expression, revealing the critical role of affinity of cis-elements in regulating the *CLV3* expression (Fig. 2, A and D). These results suggest that each cis-element contributes to *CLV3* expression and their affinities are critical to achieving proper spatial regulation.

### Single cis-elements can only activate *CLV3* at a higher WUS level

**Fig. 3.**
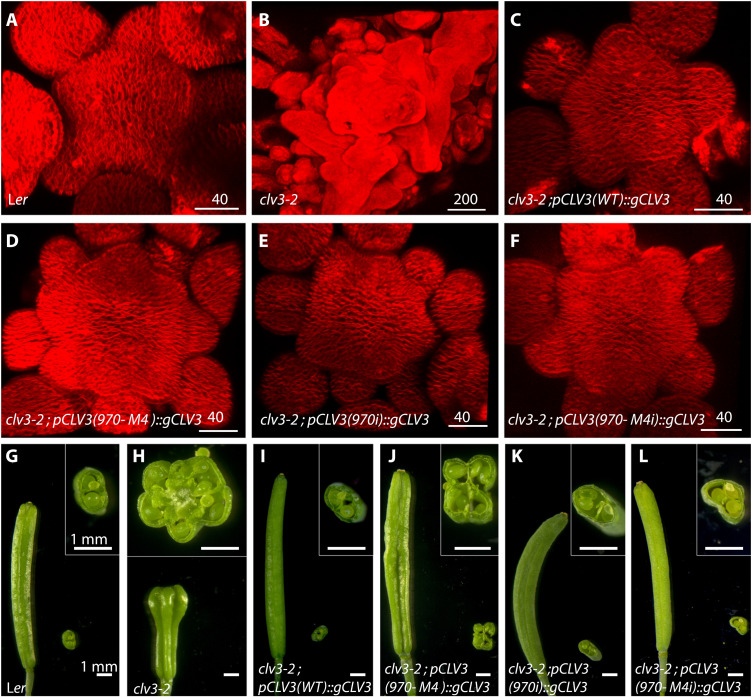
Functional analysis reveals the importance of the collective behavior of the *CLV3* CRM. (**A** to **F**) Top views of 3D-reconstructed SAMs stained with plasma membrane dye FM4-64 (red). WT (A), *clv3-2* (B), and *clv3-2* complemented with WT genomic *CLV3* (*gCLV3*) expressed from the WT *CLV3* promoter [*pCLV3(WT)::gCLV3;clv3-2*] (C), *CLV3* promoter carrying high-affinity 970M4 cis-element [*pCLV3(970M4)::gCLV3;clv3-2*] (D), CLV3 promoter carrying loss of binding mutation in 950, 997,1007, and 1060 [*pCLV3(970i)::gCLV3;clv3-2*] (E), and *CLV3* promoter carrying high-affinity mutation 970M4 and loss of binding mutations in 950, 997, 1007, and 1060 [*pCLV3(970M4i)::gCLV3;clv3-2*] (F). (**G** to **L**) Side views of intact siliques and cross section of sliced siliques. Insets show a higher-magnification view of the cross section of the sliced siliques. WT (G), *clv3-2* (H), and [*pCLV3(WT)::gCLV3;clv3-2*] (I), [*pCLV3(970M4)::gCLV3;clv3-2*] (J), [*pCLV3(970i)::gCLV3;clv3-2*] (K), and [*pCLV3(970M4i)::gCLV3;clv3-2*] (L). Scale bars (in micrometers) are given on individual panels in (A) to (F), and the scale bars in (G) to (L) are 1 mm.

The subtle changes observed upon mutating individual lower-affinity cis-elements ruled out a simple additive interaction in regulating *CLV3* expression. Therefore, to further understand the nature of interactions among cis-elements, we first determined the contribution of each one of the five cis-elements to *CLV3* expression, referred to as the intrinsic (i) behavior. We generated a library of five mutant *CLV3* reporters; each contained only one functional cis-element referred to as 970i, 997i, 1007i, 950i, and 1060i. The reporter expression analysis revealed a marked down-regulation of *CLV3* expression in outer cell layers of CZ, including the higher-affinity cis-element 970i (Fig. 2, E and F, and fig. S1). To test further the importance of affinities in influencing intrinsic behavior, we analyzed the expression of 970M4i (Fig. 2, E and H). The 970M4 cis-element is a mutation in 970 that binds WUS with three times higher affinity, and it has been shown to repress *CLV3* expression even at lower WUS in outer cell layers of CZ (*24*). The 970M4i reporter (Fig. 2, E and H, and fig. S1) was expressed at a notably higher level than 970M4 (Fig. 2, A and D). To further test whether the reactivation of *CLV3* associated with 970M4i is functionally relevant, we examined its ability to complement *clv3-2* null mutants by expressing *CLV3* genomic version. The 970M4 mutants partially complement the SAM and the floral meristem (FM) phenotypes when compared to the wild-type *CLV3* promoter (Fig. 3 and fig. S2). However, 970M4i was able to significantly better complement both the SAM and FM phenotypes, showing the reactivation of 970M4i (Fig. 3, F and L). Furthermore, both 970i (Fig. 3, E and K) and 970M4i (Fig. 3, F and L) complemented *clv3-2* to a similar extent despite binding WUS with different affinities. Consistent with this conclusion, all single cis-elements irrespective of large differences in their WUS binding affinities largely activated *CLV3* only in the inner layers of RM, where WUS accumulates at a higher level (fig. S3). However, cis-element affinity is important in the context of other functioning cis-elements in the CRM, as exemplified by the repression of 970M4. In summary, the affinity-dependent collective WUS binding to all five cis-elements is required for balancing activation and repression of transcription in regulating the spatial expression and levels of *CLV3*.

### Description of a stochastic single-cell model of *CLV3* transcription

**Fig. 4.**
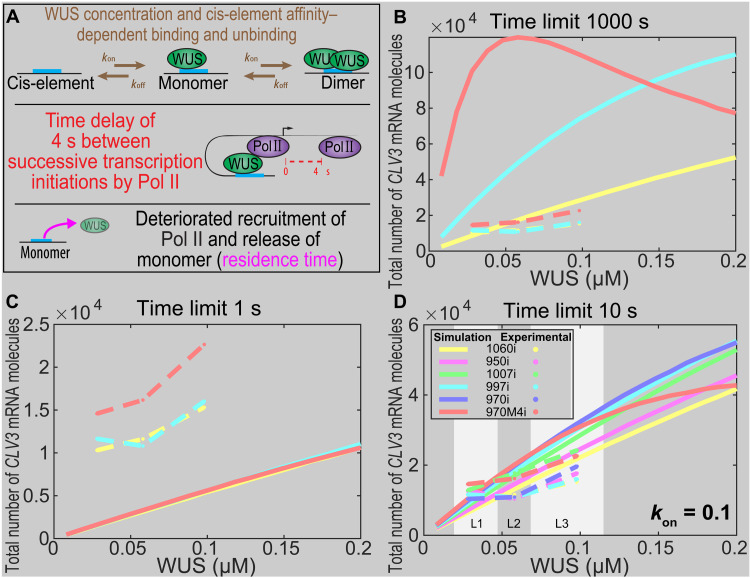
WUS protein time limit on cis-elements determines the *CLV3* levels and domain of expression. (**A**) WUS concentration–dependent binding [*k*_on_WUS], *k*_on_ is the association rate, and cis-element affinity–dependent unbinding [*k*_off_ = *K*_d_*k*_on_] determine three possible WUS occupancy states: unbound (zero WUS), monomer bound (one WUS), and dimer bound (two WUS). We assume that only the monomer bound is able to recruit RNA Pol II. A 4-s gap between recruitment of successive Pol II molecules was estimated from the Pol II elongation rate and the size of Pol II footprint on the DNA. In addition, multiple rounds of Pol II recruitment by WUS monomer deteriorate the ability of WUS to recruit additional Pol II (residence time limit). (**B** to **D**) Single-cell model of WUS-mediated activation of *CLV3* from single cis-element promoters (four mutated and only one functional cis-element). (B to D) Scaled simulation results of highest-affinity (970M4i), intermediate-affinity (997i), and lowest-affinity (1060i) cis-elements with the residence time limit of 1000 s (similar expression pattern as without the time limit since the time limit is extremely large) (B), 1 s (C), or 10 s (D). (D) All five cis-elements in addition to 970M4i and approximate WUS concentration range to reflect the corresponding WUS fold changes from L1, L2, and L3 layers.

To investigate the mechanisms of interaction among five cis-elements, we developed a stochastic modeling framework to simulate the WUS binding to the *CLV3* CRM in a single cell, together with the RNA Pol II recruitment and *CLV3* mRNA synthesis (Fig. 4A). The model was applied to understand the mechanisms underlying the *CLV3* activation by the individual cis-elements that bind WUS with different affinities and the interactions among multiple cis-elements in regulating the *CLV3* expression together. The stochasticity was introduced by implementing the Gillespie algorithm (*35*) to simulate all possible WUS binding and unbinding events to form a monomer or dimer and recruitment of Pol II for activating *CLV3* transcription. A sufficiently long time was allowed for all the simulations to reach the steady state. The *CLV3* reporter analysis performed in the wild-type background uses a steady-state WUS gradient to quantify the effects of the number, affinity, and intervening distance between cis-elements on *CLV3* expression. Since the focus of this study is to analyze concentration-dependent binding of WUS to the *CLV3* CRM, the feedback regulation of CLV3 on WUS was disabled to maintain a constant WUS concentration gradient throughout simulations to match the reporter analysis. It was also assumed that WUS binding the *CLV3* CRM alone would not change the overall WUS concentration. The stochastic time step and index for the next occurring event were generated by following the original Gillespie algorithm based on the assumption that binding to one cis-element was independent of the other cis-elements unless cooperativity among cis-elements exists. The average amount of *CLV3* mRNA synthesized, at a fixed WUS concentration, from multiple simulations was calculated. The model was then applied to measure the total amount of *CLV3* mRNA synthesized at different WUS concentrations (see the Supplementary Materials for details).

### Modeling WUS binding to the CRM

Our previous analysis revealed that each cis-element binds WUS at different concentrations as monomers first and then switches to forming dimers at increasing concentrations (*24*). Therefore, we first aimed to determine the binding and unbinding probabilities associated with each cis-element by reproducing the ratio of monomer- and dimer-bound cis-elements observed in electrophoretic mobility shift assay (EMSA) experiments (*24*). Since increasing the TF concentration decreases the search time of its binding to cis-elements (*36*), it was assumed that the probability of WUS binding to cis-elements increases with the increase in WUS concentration. In particular, the propensity of WUS binding to an empty cis-element or with a monomer is assumed to depend linearly on WUS concentration, i.e.,$k_{\text{on}}^{M}[\text{WUS}]$, where $k_{\text{on}}^{M}$ is the binding rate. Then, the unbinding propensity $k_{\text{off}}^{M}$ of WUS associated with each cis-element is calculated as $k_{\text{off}}^{M}$ = $K_{d}^{M}k_{\text{on}}^{M}$, where $K_{d}^{M}$, the dissociation constant, was quantified in our previous work (table S1) (*24*). To test this assumption, we considered a wide range of WUS concentration that encompasses WUS monomer and dimer binding to each one of the five cis-elements observed in EMSA experiments (*24*). We first simulated WUS monomer binding to a single cis-element to determine $k_{\text{on}}^{M}$, a free parameter, such that proportions of bound monomers obtained in the model were similar to those observed in the EMSA experiments with WUS that lacked the C-terminal homodimerization domain (HOD) (fig. S4A). Since dimerization occurs through sequential recruitment of WUS to the WUS monomer-DNA complex, we next modeled the dimer formation by recruiting the second WUS molecule to a monomer. In the absence of the experimental values on binding affinity associated with the WUS dimerization, we chose $K_{d}^{D}$ associated with the binding of the second WUS molecule to be the same as the one used to simulate monomer $K_{d}^{M}.$ We chose $k_{\text{on}}^{D}$ for dimer binding such that proportions of monomer and dimer bound to the cis-elements matched the EMSA experiments with full-length WUS (fig. S4B) (*24*).

### Modeling *CLV3* transcription

We considered the recruitment of Pol II as another stochastic event in the model. It has been shown that the transition from monomer binding to dimer binding could be correlated to the transcriptional switch from activation to repression of *CLV3*. Therefore, we assumed that monomer binding recruits Pol II to activate *CLV3* transcription, while the WUS dimers fail to recruit Pol II and activate *CLV3* transcription. We introduced a time delay between two successive Pol II recruitment events due to the size of the Pol II complex occupying the transcription start site. The time delay calculated based on an 80–base pair (bp) footprint of RNA polymerase and mRNA elongation rate, which is estimated to be 1.2 kb/min (*37*), was approximated as $80\;\text{bp}\times \frac{60\;s}{1200\;\text{bp}}=4\;s$. It is also assumed that, after transcription initiation, the WUS monomer can unbind or bind another WUS molecule to form a dimer. Moreover, we considered the Pol II recruitment rate as an uncalibrated parameter and carried out perturbations to examine its effect on the transcriptional output. The model was calibrated over a wide range of WUS concentrations. We then applied the model to simulate WUS binding/unbinding to a single cis-element and Pol II recruitment to generate the intrinsic expression of *CLV3* at different WUS concentrations. By comparing the *CLV3* mRNA production with the experimental quantification of the *CLV3* expression in Fig. 2 (E to H), an optimal scale of WUS concentrations was obtained to capture the WUS gradient in different cell layers of the SAM. This optimal WUS concentration scale was used in all single-cell simulations to investigate possible mechanisms controlling the intrinsic behavior of each cis-element in regulating the *CLV3* expression.

### Mechanisms of the intrinsic behaviors of cis-elements in regulating *CLV3*

It has been observed that the transcriptional output depends on the affinity of cis-elements and the TF concentration (*30*, *32*, *38*–*45*). In general, a higher-affinity cis-element results in a longer TF occupancy than the lower-affinity cis-element at a given WUS concentration. Consequently, a longer TF occupancy leads to a higher mRNA production (*46*). Experiments reveal that WUS binds to 970M4i with approximately 21.4 times higher affinity than to the lowest-affinity cis-element 1060i. Therefore, a longer residence time of WUS on 970M4i was expected to produce much higher levels of *CLV3* than 1060i. However, our experiments revealed that, although five cis-elements bound WUS with different affinities, intrinsically (950i, 970i, 997i, 1007i, and 1060i mutants), they were able to similarly activate *CLV3* only in inner cell layers of RM where the WUS concentration is higher (Fig. 2, E to H). The initial attempt in modeling by assuming WUS occupancy based on affinities produced distinct *CLV3* expression patterns for the highest 970M4i cis-element and the lowest 1060i cis-element (Fig. 4B). 970M4i produced a much sharper increase in *CLV3* expression at the lower WUS concentration than did 1060i. With the increase in WUS concentration, 970M4i produced a lower amount of *CLV3* mRNA, which is expected because of the WUS dimerization, while 1060i continued to yield higher *CLV3* mRNA (Fig. 4B). Such markedly different *CLV3* expression patterns produced by 970M4i and 1060i were not consistent with experimental observations, suggesting that additional mechanisms may regulate the intrinsic activation behavior of cis-elements in addition to their affinities.

**Fig. 5.**
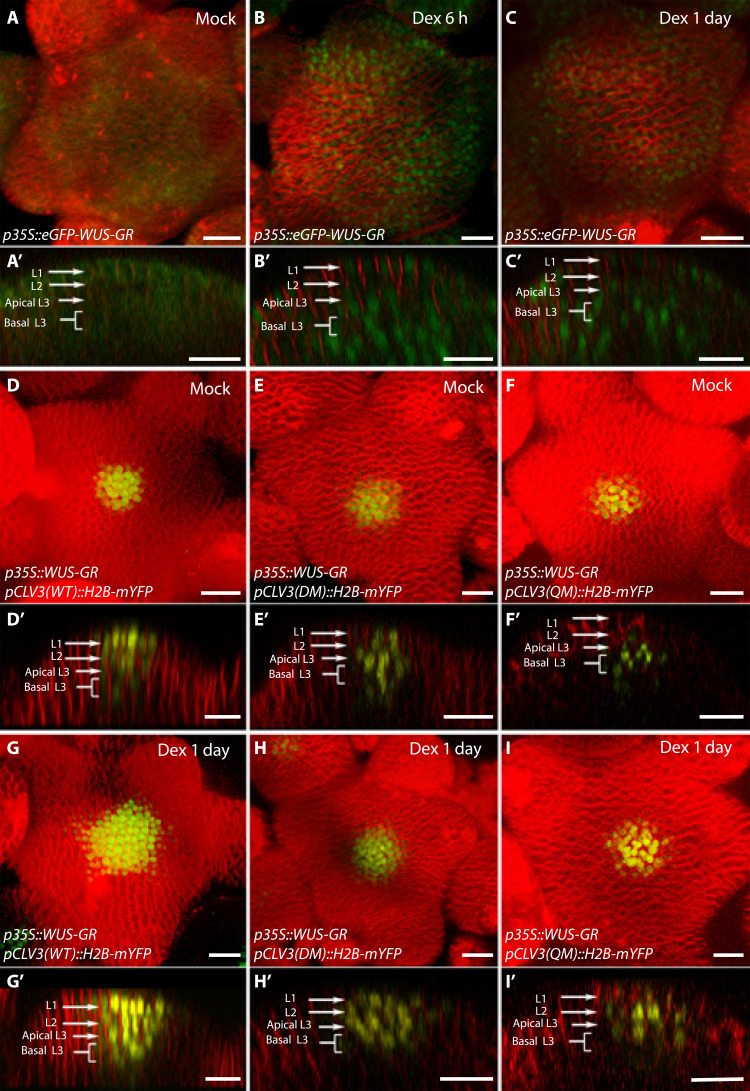
The number of cis-elements determines the sensitivity of *CLV3* promoter to the dynamic changes in the WUS protein accumulation. (**A** to **C**) SAMs showing WUS protein accumulation patterns (*p35S::eGFP-WUS-GR*) upon its Dex-induced nuclear translocation at 6 hours (B), at 24 hours (C), and upon mock treatment (A). (**D** to **I**) *p35S::WUS-GR*–expressing SAMs showing *pCLV3::H2B-mYFP* reporter expression of WT *CLV3* promoter (D), the double mutant promoter (970 and 997 mutants) (E), and the quadruple mutant promoter (970, 997, 950, and 1060 mutants) (F) upon mock treatment. The *pCLV3* reporter expression of the corresponding genotypes after 24-hour Dex treatment is shown in (G) to (I). (A to I) Three-dimensional reconstructed top views of SAMs and corresponding side views shown in (A′) to (I′). Plasma membrane stain–FM4-64 (red), eGFP-WUS-GR (green), and H2B-mYFP (yellow). Scale bars, 20 μm.

It has been noticed that for different types of TFs, including general control TF (GCN4) in yeast (*47*) and transcriptional coactivator NPR1 involved in systemic acquired resistance (SAR) in *Arabidopsis* (*48*), a higher turnover of TFs leads to a higher transcriptional activation. Furthermore, the transcriptional activation domains of GCN4 and other TFs have been shown to overlap with degradation domains, suggesting a possible correlation between transcriptional activation and TF turnover (*47*, *49*). Moreover, transcription-dependent degradation has been shown for sterol regulatory element–binding protein (SREBP) family of TFs (*50*). These observations suggest that TFs when actively transcribing may get progressively modified (for example, phosphorylated) and become transcriptionally ineffective and marked for their degradation (*47*, *49*). Although deep mechanistic links between WUS, protein phosphorylation, and protein destabilization machinery are still unknown, our earlier work suggests similarities between WUS and TFs described above. (i) The transcriptional regulatory domains [WUS-box and EAR-like (ethylene-responsive element binding factor associated amphiphilic repression) domains] function as degrons (*51*, *52*). (ii) *CLV3* activated at the lower WUS concentration in the CZ can be repressed by enriching and stabilizing the WUS protein in the nucleus (*24*, *51*). (iii) The dexamethasone (Dex)–mediated nuclear translocation of WUS by using the 35S::eGFP-WUS-GR system led to an immediate destabilization of the protein in the CZ within 6 hours (*53*). By 24 hours of Dex application, the protein was only detected in the nuclei of cells in the edge of the peripheral zone (PZ) and deeper cell layers of the RM. The *CLV3* activation and expansion into the PZ followed the centripetal pattern of rapid destabilization of the WUS protein [Fig. 5; (*53*)].

Perhaps degradation of WUS decreases the dimer concentration or creates a dynamic WUS that works favorably with the Pol II binding limit to increase *CLV3* activation. Therefore, we considered an upper limit on the residence time of WUS beyond which WUS becomes inactive and fails to recruit Pol II, referred to as residence time limit in the model (Fig. 4A). The older/inactive WUS species need to be replaced with newly synthesized WUS monomers to maintain transcription. Therefore, we imposed the same WUS monomer residence time limit for all cis-elements. A markedly lower WUS monomer residence time limit substantially decreased *CLV3* expression for all cis-elements (Fig. 4C and fig. S5). Simulations with a balanced residence time limit were able to generate a similar intrinsic expression pattern of *CLV3* for all cis-elements. In particular, to generate similar expression patterns of 970M4i (highest affinity) and 1060i (lowest affinity) cis-elements, we chose the residence time limit to be 10 for all simulations involving multiple cis-elements discussed in the following sections (Fig. 4, B to D, and fig. S5).

### The *CLV3* CRM composition determines sensitivity to dynamic changes in WUS protein levels

The number of cis-elements may also determine the sensitivity of the *CLV3* promoter to WUS levels to regulate spatial expression of *CLV3*. To test this, we analyzed the response of the mutant promoters lacking several WUS binding cis-elements to *35S::eGFP-WUS-GR* system, upon 24 hours of Dex application, described in the previous section. The wild-type *CLV3* promoter with five functional cis-elements expressed at high levels and the promoter activity expanded into the PZ (Fig. 5, D and G). The mutant promoter lacking the two functional WUS binding cis-elements (970M and 997M)-*pCLV3(DM)::H2b-mYFP* is initially expressed in the deeper cell layers, and the expression levels are below that of the wild-type promoter (Fig. 5E). The 24-hour Dex application was able to activate *pCLV3(DM)* in the CZ weakly but failed to expand into the PZ (Fig. 5H) (*n* = 8) when compared to the wild-type promoter, which revealed strong activation and radial expansion (Fig. 5G). The mutant promoter lacking four cis-elements (950M, 970M, 997M, and 1060M)-*pCLV3(QM)::H2b-mYFP* was expressed only in the deeper layers (Fig. 5F). After 24 hours of Dex application, the mutant promoter was mildly up-regulated in deeper layers; however, it failed to activate in the CZ and expand radially into the PZ (Fig. 5I). Together, rapid destabilization of WUS can lead to higher *CLV3* activation, which is maintained even at undetectable WUS protein levels, showing that all five cis-elements working together increase the sensitivity of *CLV3*.

### Cooperativity among cis-elements regulates *CLV3* expression

**Fig. 6.**
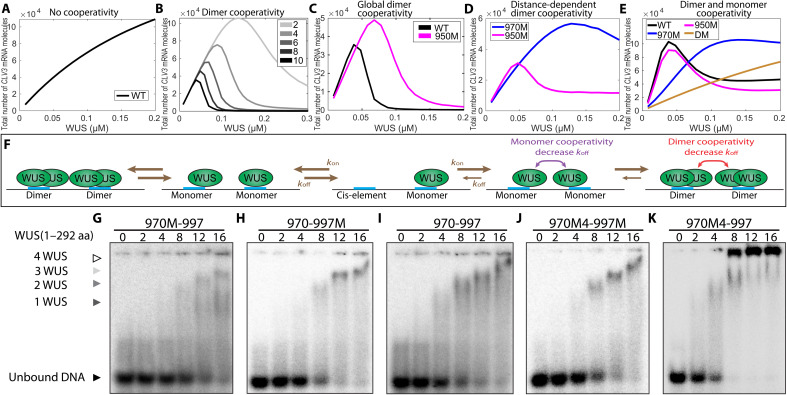
Cooperativity among cis-elements regulates *CLV3* expression. Average *CLV3* mRNA levels from single-cell simulations in response to WUS concentration without cooperativity (**A**), at different strengths of dimer cooperativity (**B**), when dimer cooperativity between every cis-element is considered (**C**), when the dimer cooperativity depends on the intervening distance between cis-elements (**D**), and when both WUS monomer and dimer cooperativity were considered (**E**). (**F**) Binding and unbinding dynamics of WUS monomer and dimer on cis-elements. (**G** to **K**) Gel shift assay of increasing concentrations of full-length WUS 1 to 292 amino acids (aa)] to probes of similar length that cover the 970 and 997 cis-elements. Probes with loss of binding mutations to the TAAT elements in the 970 cis-element (G) and the 997 cis-element (H). (I) Probe with WT copies of the 970 and 997 cis-elements. Probes that contain higher-affinity mutant 970M4 along with the mutant 997 (J) or WT 997 cis-element (K). (G to K) Arrowheads denote higher-order WUS complexes: monomer (dark gray), dimer (light gray), and higher complexes (white). The unbound probe (black).

Our experimental analysis showing different expression patterns of *CLV3* for single cis-elements and multiple cis-elements suggested an interaction among cis-elements within the CRM (*24*). The same study also showed that an increase in cis-element affinity (970M4) resulted in a decrease in dimerization threshold and repressed *CLV3* in outer cell layers of CZ where WUS accumulates at a lower level. These observations suggested that cis-element affinity is important in the context of the multiple cis-elements, possibly in inducing cooperative interactions among WUS dimers bound to multiple cis-elements within the CRM. To understand the multiple cis-element behaviors, we used the calibrated single-cell WUS binding model by extending it to include multiple cis-elements. Without any cooperative interactions among them, an increase in WUS concentration led to an increase in *CLV3* expression, which can be interpreted as a linear combination of intrinsic behaviors of individual cis-elements, which is not consistent with the experimental analysis (Fig. 6A). Therefore, we introduced cooperativity among cis-elements into the model. First, we considered equal cooperativity among all cis-elements irrespective of the intervening distance. As the cooperativity increased, the *CLV3* expression decreased at the higher WUS concentration, which could be due to increased dimerization (Fig. 6B). Then, we chose appropriate values for parameters involved in the dimer cooperativity to obtain the highest activation of *CLV3* at a lower WUS concentration as observed in experiments. Next, we used the calibrated model with the chosen cooperativity parameters to simulate mutant *CLV3* consisting of different number of cis-elements. In particular, our experimental analysis showed a weaker down-regulation of *CLV3* upon mutating any one of the four lower-affinity cis-elements (950M, 997M, 1007M, and 1060M) for the low WUS concentration, compared to the highest affinity, i.e., 970 cis-element (970M) (Fig. 2, A to C). However, in the simulations with the calibrated equal dimer cooperativity, 950M was expressed at a much higher level than the wild type at the high WUS concentration (Fig. 6C), which was not consistent with the experimental observation, suggesting unequal cooperativity among those cis-elements in the CRM.

Next, we introduced unequal dimer cooperativity wherein the interaction between neighboring cis-elements was higher, and cooperativity decreased with increasing intervening distance (referred to as distance-dependent cooperativity). Similar kind of cooperativity was studied in thermodynamic models earlier (*54*). For simplicity, we simulated 970M and 950M expressions representing mutations in high- and low-affinity cis-elements, respectively. The model with distance-dependent dimer cooperativity was able to generate wild-type expression patterns. However, a similar expression behavior was observed for both 950M and 970M at lower WUS concentrations, which is inconsistent with the experimental data (Fig. 6D). This suggested that the cis-element affinity influences interactions among cis-elements and the higher-affinity cis-element 970 interacts differently than the lower-affinity cis-elements in activating *CLV3* at lower WUS. Therefore, distance-dependent monomer cooperativity between cis-elements was considered. The monomer and dimer cooperativity were considered separately since one is responsible for activation and the other one is responsible for repression. Considering that the affinity plays a role when multiple cis-elements interact, the residence time limit associated with single cis-element was disabled. The additional WUS monomer cooperativity along with the dimer cooperativity between all cis-elements was able to generate expected wild-type and mutant (970M and 950M) cis-element behaviors at all WUS concentrations (Fig. 6E), showing the importance of both in regulating *CLV3* transcription.

### The neighboring cis-elements influence WUS DNA-protein complex formation

To test predictions of model simulations on the possible cooperative behavior of cis-elements, we performed EMSA with increasing concentration of WUS on probes that contain two adjacent cis-elements. We considered the two adjacent cis-elements 970 and 997 because mutating these two cis-elements has been shown to down-regulate *CLV3* expression in outer cell layers of CZ and up-regulate expression in the inner layers of RM (*24*). Full-length WUS at lower concentration has been shown to bind as a monomer to single cis-elements, which shifts to a dimeric complex at the higher WUS concentration (*24*). We found that WUS bound the oligo that contains 970 and 997 cis-elements (Fig. 6I) at much lower concentrations than observed with the oligos of the same length that only contains one functional cis-element that is either the 970 (Fig. 6H) or 997 cis-element (Fig. 6G). In addition, the WUS shifted to form higher molecular weight complexes at much lower concentrations with the two functional cis-elements than one functional cis-element (Fig. 6I). To further test the nature of the protein and complex formation across multiple cis-elements, we tested the binding patterns of two WUS protein variants: WUS1-134 that only contained the DNA binding domain and lacked the centrally located HOD, and WUS1-208 that contains the centrally located HOD domain. Our earlier work has shown that these fragments bind cis-elements with comparable affinities to the full-length protein (*24*). With increasing concentration of WUS1-134, a gradual switch from monomeric to the higher molecular complex was observed, which is expected as previous work has shown that the DNA binding domain also participates in dimerization (*24*, *29*). With WUS1-208, at the same protein concentration range, we observed a faster shift from the monomer form into the higher molecular weight complex. Testing these two protein versions on a probe containing only one functional 970 cis-element revealed higher molecular complex formation at a much higher concentration (*24*). These results suggest that the second dimerization domain may facilitate interaction between WUS molecules bound to the adjacent cis-elements in promoting higher molecular WUS complex formation.

### The distance between cis-elements is critical for *CLV3* expression

**Fig. 7.**
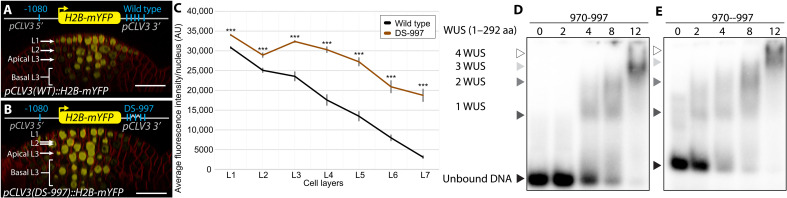
Spacing between cis-elements is critical for *CLV3* repression. Side view of SAM showing the WT *pCLV3::H2B-mYFP* expression (**A**) and mutant *CLV3* reporter containing duplicated sequence to the left (5′) and right (3′) of the 997 cis-element (double sequence around 997—DS-997) (**B**). Scale bars, 20 μm. (**C**) Average H2B-mYFP fluorescence intensity (mean ± SE) in 10 centrally located nuclei/cell layers quantified from four independent transformants of WT and DS-997 (*n* = 4). ***P < 0.001. EMSAs showing increasing concentrations of full-length WUS (1 to 292 amino acids) bound to the probe containing the 970 and 997 cis-elements with WT intervening sequence (**D**) or a duplicated intervening sequence (**E**).

The cooperativity observed in gel shift assays suggests that the neighboring cis-elements increase WUS binding, possibly through protein-protein interaction facilitated by the second HOD (HOD2). To test the influence of spacing between cis-elements without reducing the number or affinity, we duplicated the sequence between neighboring cis-elements. The increased distance might reduce the interaction of WUS bound to neighboring cis-elements without affecting the intrinsic binding to each independent cis-elements. Therefore, we duplicated the intervening sequence between 970-997 and 997-1007 (double space around 997) *pCLV3(DS-997)::H2B-mYFP*. Increasing the distance between neighboring cis-elements led to increased *CLV3* expression in all cell layers, and an increase in the deeper layers was much higher than in the outer cell layers of CZ (Fig. 7, A to C). These results suggest that the distance between cis-elements is more critical for the repression of *CLV3*, likely through the formation of large WUS complexes across neighboring cis-elements. To test whether the increased distance between 970 and 997 cis-elements alters the binding dynamics, we analyzed WUS binding to the oligo with duplicated sequences that doubled the distance between 970 and 997 (970--997). The full-length WUS protein could bind the oligo (970--997) at lower WUS (Fig. 7, D and E). However, the transition from lower molecular weight complexes to higher molecular weight complexes occurred over a much wider WUS concentration range. Therefore, the increase in *CLV3* expression in all cell layers seen in DS-997 could be explained by the larger WUS concentration range over which it remains as a lower molecular weight complex, showing that, in addition to the affinity of the cis-elements, the intervening distance is important in regulating the *CLV3* expression.

### Description of a 3D cell–based model of *CLV3* transcription

The single-cell model provided insights into the WUS binding dynamics with individual cis-elements, Pol II recruitment, and minimum cooperativity mechanisms required for *CLV3* expression (fig. S6). However, the single-cell model can only provide average expression behavior at given WUS concentrations, without considering the tissue spatial organization and the stochasticity associated with individual cells within layers of the SAM under a broader range of WUS concentrations. Therefore, we expanded our scope of study by developing a 3D multicellular model to capture the tissue-level spatial dynamics.

**Fig. 8.**
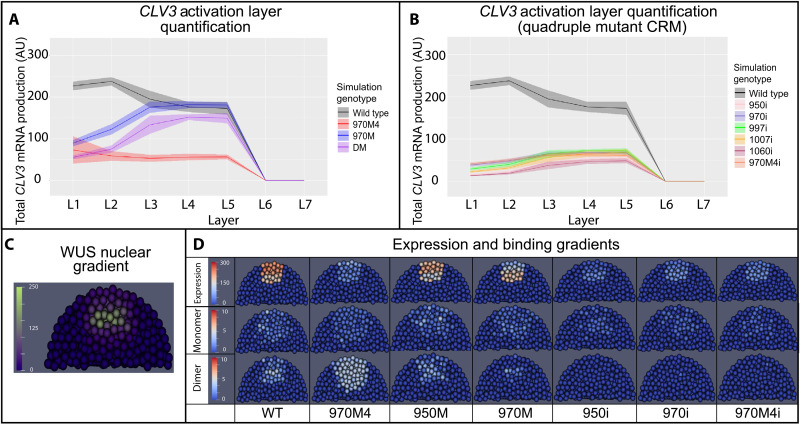
Simulated *CLV3* dynamics and WUS protein complexes in the 3D SAM model. (**A**) Levels of *CLV3* (mean = line, SD = shaded area) activation in WT system and system with selected mutated cis-elements. (**B**) Levels of *CLV3* gene activation (mean = line, SD = shaded area) in WT system and system carrying all possible combinations of quadruple mutants (reflects intrinsic behavior of each functional cis-element). Line indicates mean *CLV3* in different cell layers. Shaded area indicates SD of activation among the cells in a given cell layer. L1 to L7 indicate the layers of the SAM from outermost CZ to inner layers of the RM. (**C**) Spatial distribution of nuclear-localized WUS. (**D**) Median longitudinal sections of simulated SAMs showing WUS monomer (middle), WUS dimer (bottom), and *CLV3* expression (top) in WT and system carrying various mutant cis-elements 970M4, 950M, 970M, 950i, 970i, and 970M4i.

The 3D model could help quantify the establishment of the *CLV3* expression pattern throughout the tissue by simulating the stochastic single-cell model in individual cells simultaneously at different WUS concentrations. The 3D model was constructed on the basis of the framework used in our previous work (*27*) combined with new biological data and mechanisms identified by using the stochastic single-cell model. The computational domain consisted of a 3D matrix of unit spheres organized in a half-dome shape, corresponding to cells within the SAM from the L1 to L7 layers. At the tissue level, a spatial gradient of WUS proteins across different layers, which captured a similar fold change from deeper layers to outer layers observed in experiments (Fig. 8C and fig. S3), was introduced and maintained at this fixed concentration throughout each simulation (Figs. 1A and 8C). In individual cells, the single-cell stochastic model was applied to simulate WUS binding with cis-elements by using the local WUS concentrations to regulate *CLV3* transcription. The same mechanisms identified by the single-cell stochastic model were implemented under wild-type and multiple cis-element mutant conditions. Each simulation was allowed to run long enough to achieve the steady-state behavior, and the parameters used in the simulations are listed in table S3.

### Analysis of *CLV3* expression and WUS complexes captured by the 3D model

Using a biologically relevant WUS gradient (Fig. 8C), *CLV3* simulations were generated under a variety of different conditions, including wild type, four cis-elements (970M), three cis-elements (DM), and single cis-element (e.g., 970i). The behaviors of several cis-element mutants are shown in Fig. 8 (A and B). *CLV3* expression in wild type was generally higher than in other mutants, similar to the experimental data shown in Fig. 2A. In particular, wild-type *CLV3* activation was highest in the L1 layer and lowest in the inner layers of RM. 970M showed a higher expression in the inner layers of RM than in the outer L1 layer. Of particular interest was 970M4, in which the affinity was strengthened over the default 970 affinity, expressed at a lower level in all cell layers. When simulating the mutants with a single functional cis-element in the CRM, e.g., 950i, 970i, 997i, 1007i, and 1060i, the *CLV3* expression was detected in only the inner layers of RM. Other than the minor difference in the magnitude, all single cis-element mutants expressed only in the inner cell layers (Fig. 8B), similar to the experimental results. Simulations also showed an impairment in the spatial patterns of *CLV3* expression as more cis-elements were deleted. For example, the deletion of a single lower-affinity cis-element 950 (950M) had a relatively minimal effect on *CLV3* activation (Fig. 8D). In contrast, deletion of the higher-affinity cis-element 970 (970M) shifted *CLV3* expression to the inner layers (Fig. 8D). The more drastic shift in *CLV3* expression into deeper layers occurred when deleting four cis-elements (e.g., 950i or 970i) regardless of their WUS binding affinity (Fig. 8D). Therefore, the cooperativity mechanism identified by the single-cell stochastic model was able to generate the expected *CLV3* expression behavior in the 3D model.

Bimolecular fluorescence complementation (BiFC) assays in plants expressing split enhanced green fluorescent protein (eGFP)–WUS constructs expressed from the native *WUS* promoter revealed very few fluorescent positive cells in the L3 and L2 layers of SAMs (fig. S7). These results show that WUS dimerizes in cells that accumulate higher levels of WUS, supporting the correlation observed in biochemical analysis. However, the observed dimerization in BiFC assays does not distinguish between DNA-bound WUS complexes and unbound complexes. Moreover, it likely represents WUS complexes with cis-elements of many target genes (*27*). Therefore, we used the 3D model to visualize the spatiotemporal distributions of WUS complexes including monomers and dimers on the *CLV3* promoter across cell layers in SAMs (Fig. 8D). A higher concentration of WUS monomers in the outer layers of CZ and higher dimers in the inner layers of RM were observed for the wild-type and lower-affinity cis-element 950M. Deleting the 970 cis-element showed lower levels of WUS monomers in the outer layers of CZ and lower levels of dimers in the inner layers of RM (Fig. 8D). This suggested that the higher-affinity cis-element exerts a stronger influence on *CLV3* transcription, but it was not sufficient to completely activate in the outer layers of the CZ or repress the inner layers of RM on its own, showing that cis-elements interact with each other in maintaining specific amounts of WUS monomer and dimer complexes in different layers in regulating *CLV3* expression. The 970M4 results resolved the seemingly paradoxical expression patterns of this mutant. A massive amount of WUS dimers in all layers can explain a marked reduction of *CLV3* expression. In contrast, both monomers and dimers accumulated at a lower level when only one cis-element was functional, showing that WUS failed to populate at higher levels on cis-elements likely due to the lack of cooperativity. Overall, the 3D model simulations showed the spatial distributions of WUS complex formation at a quantitative level in different cell layers of SAMs. The WUS complex formation could be correlated to WUS concentration in different cell layers and the affinity-dependent cooperative behavior of cis-elements in expressing *CLV3* in the CZ.

### Effect of cooperativity on the spatial patterns of *CLV3* transcription

**Fig. 9.**
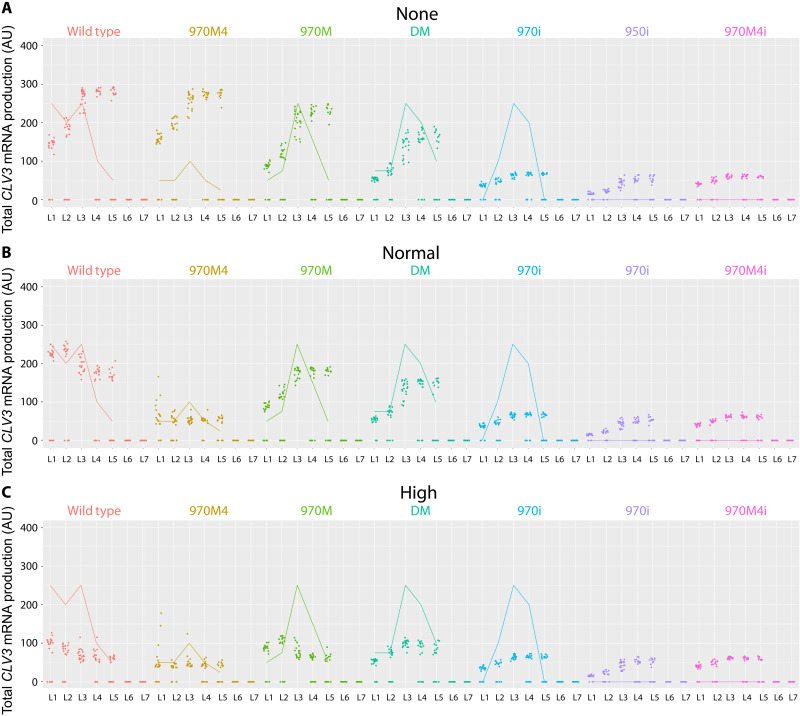
Cooperativity levels influence *CLV3* activation. *CLV3* activation level of WT *CLV3* CRM and mutant *CLV3* CRMs under various cooperativity levels. (**A**) No cooperativity: simulations had no cooperativity between cis-elements. (**B**) Normal cooperativity: normal cooperativity values (0.01 monomer cooperativity, 0.2 dimer cooperativity) used in the default simulations. (**C**) High cooperativity: simulations with 10× the cooperativity of the default values in the simulation, i.e., 0.001 monomer cooperativity and 0.02 dimer cooperativity. Dots are simulation values for a cell. Lines are corresponding average expression values from experimental studies. Colors represent different mutants.

The experimental evidence suggested that the cooperativity among cis-elements is critical to achieving proper spatial patterns of *CLV3* expression. To better understand the role of cooperativity in the robust regulation of *CLV3* expression quantitatively, we imposed different levels of cooperativity between monomers or dimers for both wild-type and mutant conditions. A complete removal of cooperativity led to a higher *CLV3* expression in the inner cell layers of RM and a lower expression in outer cell layers of CZ under all conditions (Fig. 9A). In contrast, increasing cooperativity led to *CLV3* down-regulation (Fig. 9C), showing that strength of cooperativity influences *CLV3* expression. Our experimental analysis shows that increasing the cis-element affinity (970M4) leads to down-regulation of *CLV3* expression, which could be due to a higher cooperativity among cis-elements leading to the repression. To test this hypothesis, we removed cooperativity from 970M4, which led to an increase in *CLV3* expression, and the pattern of expression resembled that of wild type (Fig. 9A). These results show the importance of cooperativity in modulating *CLV3* expression, which in turn depends on the cis-element affinity.

Our experimental analysis also showed that decreasing the number of cis-elements leads to a decrease in *CLV3* expression in outer cell layers of CZ and an increase in inner cell layers of RM, suggesting that the number of cis-elements may also aid in inducing cooperativity. Consistent with the requirement of multiple cis-elements in mediating cooperativity, the effects of cooperativity levels on *CLV3* expression diminished with the deletions of multiple cis-elements (Fig. 9).

**Fig. 10.**
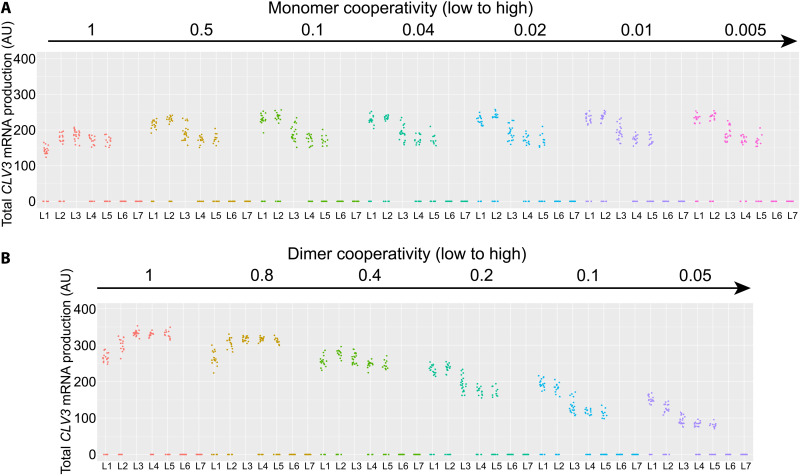
Independent perturbations of the monomer or dimer cooperativity. The effect of changes in monomer cooperativity (from 1 to 0.005) and dimer cooperativity (from 1 to 0.05) on *CLV3* activation. The direction of the arrows indicates an increase in cooperativity. In row (**A**), dimer cooperativity was held constant at 0.2, while monomer cooperativity was varied (1 to 0.005). In row (**B**), monomer cooperativity was held at 0.01 and dimer cooperativity was varied (1 to 0.05). A complete table of changes in monomer and dimer cooperativity is presented in fig. S6. The individual graphs represent the *CLV3* activation in different cell layers (L1 to L7) of simulated SAMs under the cooperativity levels noted for each simulation. The dots represent the values of the *CLV3* signal for individual simulated cells.

As shown above, removing the overall cooperativity that includes both the monomer and dimer cooperativity leads to the internalization of *CLV3*, which is not entirely consistent with the in vivo observed overall increase of *CLV3* expression even in the outer cell layers of *pCLV3(DS-997)* (Fig. 7, B and C). Removing the overall cooperativity that also included the monomer cooperativity might have caused the down-regulation of *CLV3* in outer cell layers of CZ. Therefore, we perturbed monomer and dimer cooperativity independently. At a constant dimer cooperativity, increasing monomer cooperativity alone led to a gradual increase in *CLV3* expression in outer cell layers and expression maxima shifted to outer cell layers (Fig. 10A and figs. S8 and S9). In contrast, increasing the dimer cooperativity alone led to an overall decrease in *CLV3* expression, which was more pronounced in the inner layers of RM and a shift in the expression maxima to the outer layers of CZ (Fig. 10B and figs. S8 and S9). This suggests that *CLV3* expression is regulated through a balance between dimer and monomer cooperativity mediating the repression and activation, respectively. These simulation results could also help us to understand the experimental data, in which the increased expression of *CLV3* in all cell layers observed upon doubling the distance (DS-997) could be attributed to lower dimer cooperativity leading to derepression. Together, these results show that cooperativity plays a critical role in regulating *CLV3* expression when all five cis-elements are functional.

## DISCUSSION

A homotypic cluster of five cis-elements with different WUS binding affinities regulates levels and spatial expression of *CLV3*. WUS has been shown to activate and repress *CLV3* at lower and higher levels, respectively. Our work reveals that the relative affinities of each element, the number of cis-elements, and intervening distance contribute to the collective effect. Moreover, the collective activity of the CRM arises not only because of the individual affinity but also because of cooperative binding of multiple neighboring cis-elements to WUS. WUS was previously shown to form a mixture of monomers, dimers, and oligomers in solution over a wide concentration range (*24*). Moreover, DNA/cis-elements have been shown to promote dimerization or multimerization of WUS over a small two- to fourfold increase in the WUS level.

Our biochemical analysis presented here reveals that two adjacent cis-elements can increase the binding sensitivity of WUS at lower levels than the single cis-elements, suggesting that the cis-elements cooperate in increasing the binding probability of WUS monomers, which could contribute to boost activation. Our biochemical work also shows that the two cis-elements working together allows the formation of higher-order WUS complexes at lower WUS levels, which depends on the second HOD (Fig. 6, J and K). This suggests that the second HOD may allow interaction of WUS species bound to the adjacent cis-elements in forming higher-order complexes. WUS has two dimerization domains, one of which is near the DNA binding domain and the other is found outside the DNA binding domain (*24*). The second dimerization domain may allow protein-protein interaction across neighboring cis-elements, which then allows cooperative binding across the cis-elements. Our analysis also reveals that cis-element affinity plays a critical role in inducing cooperativity across cis-elements. The increased affinity of 970M4 cis-element contributed to higher cooperativity, leading to the repression of *CLV3*. However, such repression requires other functional cis-elements in the CRM, showing that the collective behavior arises as a result of the number of cis-elements and the WUS binding affinities. The collective behavior of a low-affinity homotypic CRM has been shown to be critical in a recent study of the *Drosophila* SHAVENBABY locus. Increasing the binding affinity of one of the cis-elements resulted in a strong ectopic activation, suggesting that low-affinity homotypic CRMs may lead to higher specificity (*9*). Our work showing the importance of the number of cis-elements in regulating gene expression agrees with the fundamental concept of having multiple cis-elements organized in a constellation leading to gene expression specificity. However, the *CLV3* CRM regulation differs from other homotypic CRMs such as SHAVENBABY locus where *CLV3* expression is regulated through a concentration-dependent activation-repression switching mechanism. The C terminus of WUS has been shown to bind at least three proteins: HAIRYMERISTEM (*55*), SHOOT-MERISTEMLESS (*56*), and TOPLESS (*57*). Earlier analysis shows that the C terminus of WUS is not required for the regulation of DNA binding affinity and dimerization (*24*) and DNA binding specificity (*29*). Therefore, we suggest that WUS binding to the *CLV3* CRM is a cofactor-independent mechanism that depends on the organization of cis-elements in the CRM. Besides *CLV3*, WUS has been shown to activate and repress several hundred genes (*27*). Our bioinformatics search for “TAAT” core-containing cis-element clusters (see the Supplementary Materials for details of the algorithm) identified multiple clusters in 152 of 154 WUS up-regulated genes and 298 of 303 WUS down-regulated genes (tables S6 and S7). This resource should guide future in vivo analysis to refine our understanding of the relationship between CRMs and gene expression specificity.

Our analysis also shows that the interaction between cis-elements in promoting higher molecular WUS complexes also depends on the distance between cis-elements. Increasing the distance between cis-elements unexpectedly decreased the WUS detection threshold, suggesting that distance may also play a role in sensing WUS concentration through an unknown mechanism. This might increase the probability of WUS monomer binding to adjacent cis-elements. However, the stabilization of WUS into a higher molecular weight complexes still occurred at the same WUS levels as observed with the wild-type distance. Thus, the increase in *CLV3* expression observed upon increasing the distance could be due to increased activation and not entirely due to the reduced repression. Together, our results show that the cis-element affinity plays a dominant role in *CLV3* repression, while it appears that the system can withstand an increase in intervening distance in forming higher WUS complexes.

The computational model developed in this study allows us to recreate and, in a sense, verify the plausibility of our mechanistic explanations of experimental results. It was possible to quantify properties that are very difficult to obtain through experimental means such as the residence time of WUS on cis-elements to calibrate the model and visualization of concentration-dependent ratios of WUS monomer and dimer/higher-order complexes on the *CLV3* cis-elements. The upper limit on the residence time of WUS was critical to explain individual cis-element behaviors that differ in their binding affinities. Our experimental analysis shows that a higher WUS turnover leads to a higher *CLV3* activation, suggesting that older WUS species may become ineffective and may unbind. The nuclear export of WUS has been shown to play a crucial role in regulating the WUS nuclear concentration (*51*). It has also been shown that a nuclear export signal is required for WUS degradation in the cytoplasm. Perhaps the older WUS molecules that unbind are exported and degraded in the cytoplasm, which may create space for newly synthesized WUS that moves into the outer layer of CZ to bind cis-elements to sustain *CLV3* activation. CLV3 has been shown to offset nuclear export of WUS, which forms an additional feedback mechanism in regulating the nuclear concentration (*51*). Whether CLV3 levels also independently determine residence time of WUS by influencing its unbinding from cis-elements perhaps by regulating the WUS protein modifications remains to be explored. Nevertheless, a seamless connection involving WUS binding, unbinding, export, and degradation could lead to a robust maintenance of *CLV3* transcription. However, the current model assumes a constant WUS gradient and is limited to exploring the mechanisms underlying the *CLV3* expression without considering the feedback regulations of CLV3 signaling on WUS. Our recent study developed a model involving both transcriptional and posttranslational regulations of WUS by the CLV3 signaling (*51*). This model used a generic function of WUS concentration to represent the *CLV3* transcription. The model perturbations revealed that the dual control of *WUS* transcription and nuclear levels by the CLV3 signaling when coupled to the WUS concentration–dependent transcriptional activation and repression of *CLV3* leads to a robust maintenance of the WUS protein gradient. Our results show that the cis-element mutant reporter 970i was markedly reset into the outer layers of CZ in the *clv3* null mutants complemented with the 970i genomic construct (fig. S10, A and B). Perhaps this is due to the effects of altered CLV3 signaling on the expression and nuclear accumulation of WUS establishing a new gradient. In the future, coupling the 3D stochastic model of *CLV3* transcription developed here with the CLV3 signaling model of the regulation of WUS transcription and the WUS protein dynamics should allow assessment of the influence of different properties of the *CLV3* CRM, including the number of cis-elements in regulating the robustness of the WUS gradient.

## MATERIALS AND METHODS

### Experimental design

Plants were grown under continuous light as described earlier in (*24*). Imaging was performed on the Zeiss 880 AIRYSCAN upright under a 40× objective. eGFP-WUS was excited at 488 nm and collected with filter 495 to 550 nm. Histone 2B modified yellow fluorescent protein (H2B-mYFP) was excited at 514-nm filtered with main beam splitter (MBS) 458/514/561/633 and collected with band-pass (BP) filter 495 to 550 nm. FM4-64 was excited at 561 nm and collected with BP 570 to 620 nm.

### Stochastic single-cell model and the 3D cell–based model

Description of two computational models developed in this study is provided in detail in the Supplementary Materials. Parameters used in the stochastic single-cell model can be found in tables S1 and S2. Parameters used in the 3D cell-based model can be found in tables S1 and S3.

### Statistical analysis

The source data associated with all experiments are presented in the additional data files. In addition, the means, *N*, and *P* values are included within each dataset.

## Supplementary Materials

### This PDF file includes:

Supplementary Text Figs. S1 to S11 Tables S1 to S5 References

## Notes

View/request a protocol for this paper from *Bio-protocol*.

## References

[1] M. Levine, Transcriptional enhancers in animal development and evolution. Curr. Biol. 20, R754–R763 (2010). 20833320 10.1016/j.cub.2010.06.070 PMC4280268

[2] C.-T. Ong, V. G. Corces, Enhancer function: New insights into the regulation of tissue-specific gene expression. Nat. Rev. Genet. 12, 283–293 (2011). 21358745 10.1038/nrg2957 PMC3175006

[3] F. Spitz, E. E. M. Furlong, Transcription factors: From enhancer binding to developmental control. Nat. Rev. Genet. 13, 613–626 (2012). 22868264 10.1038/nrg3207

[4] J. Banerji, S. Rusconi, W. Schaffner, Expression of a beta-globin gene is enhanced by remote SV40 DNA sequences. Cell 27, 299–308 (1981). 6277502 10.1016/0092-8674(81)90413-x

[5] M. Slattery, T. Riley, P. Liu, N. Abe, P. Gomez-Alcala, I. Dror, T. Zhou, R. Rohs, B. Honig, H. J. Bussemaker, R. S. Mann, Cofactor binding evokes latent differences in DNA binding specificity between Hox proteins. Cell 147, 1270–1282 (2011). 22153072 10.1016/j.cell.2011.10.053 PMC3319069

[6] G. Struhl, K. Struhl, P. M. Macdonald, The gradient morphogen *bicoid* is a concentration-dependent transcriptional activator. Cell 57, 1259–1273 (1989). 2567637 10.1016/0092-8674(89)90062-7

[7] W. D. Fakhouri, A. Ay, R. Sayal, J. Dresch, E. Dayringer, D. N. Arnosti, Deciphering a transcriptional regulatory code: Modeling short-range repression in the Drosophila embryo. Mol. Syst. Biol. 6, 341 (2010). 20087339 10.1038/msb.2009.97 PMC2824527

[8] R. Joshi, J. M. Passner, R. Rohs, R. Jain, A. Sosinsky, M. A. Crickmore, V. Jacob, A. K. Aggarwal, B. Honig, R. S. Mann, Functional specificity of a Hox protein mediated by the recognition of minor groove structure. Cell 131, 530–543 (2007). 17981120 10.1016/j.cell.2007.09.024 PMC2709780

[9] J. Crocker, N. Abe, L. Rinaldi, A. P. McGregor, N. Frankel, S. Wang, A. Alsawadi, P. Valenti, S. Plaza, F. Payre, R. S. Mann, D. L. Stern, Low affinity binding site clusters confer Hox specificity and regulatory robustness. Cell 160, 191–203 (2015). 25557079 10.1016/j.cell.2014.11.041 PMC4449256

[10] B. P. Berman, Y. Nibu, B. D. Pfeiffer, P. Tomancak, S. E. Celniker, M. Levine, G. M. Rubin, M. B. Eisen, Exploiting transcription factor binding site clustering to identify cis-regulatory modules involved in pattern formation in the Drosophila genome. Proc. Natl. Acad. Sci. U.S.A. 99, 757–762 (2002). 11805330 10.1073/pnas.231608898 PMC117378

[11] A. P. Lifanov, V. J. Makeev, A. G. Nazina, D. A. Papatsenko, Homotypic regulatory clusters in Drosophila. Genome Res. 13, 579–588 (2003). 12670999 10.1101/gr.668403 PMC430164

[12] S. Small, A. Blair, M. Levine, Regulation of even-skipped stripe 2 in the Drosophila embryo. EMBO J. 11, 4047–4057 (1992). 1327756 10.1002/j.1460-2075.1992.tb05498.x PMC556915

[13] Y. T. Ip, R. E. Park, D. Kosman, E. Bier, M. Levine, The dorsal gradient morphogen regulates stripes of rhomboid expression in the presumptive neuroectoderm of the Drosophila embryo. Genes Dev. 6, 1728–1739 (1992). 1325394 10.1101/gad.6.9.1728

[14] W. Driever, C. Nüsslein-Volhard, The bicoid protein is a positive regulator of *hunchback* transcription in the early *Drosophila* embryo. Nature 337, 138–143 (1989). 2911348 10.1038/337138a0

[15] J. Gaudet, S. E. Mango, Regulation of organogenesis by the *Caenorhabditis elegans* FoxA protein PHA-4. Science 295, 821–825 (2002). 11823633 10.1126/science.1065175

[16] J. Jiang, M. Levine, Binding affinities and cooperative interactions with bHLH activators delimit threshold responses to the dorsal gradient morphogen. Cell 72, 741–752 (1993). 8453668 10.1016/0092-8674(93)90402-c

[17] S. Rowan, T. Siggers, S. A. Lachke, Y. Yue, M. L. Bulyk, R. L. Maas, Precise temporal control of the eye regulatory gene Pax6 via enhancer-binding site affinity. Genes Dev. 24, 980–985 (2010). 20413611 10.1101/gad.1890410 PMC2867212

[18] L. Wolpert, Positional information and the spatial pattern of cellular differentiation. J. Theor. Biol. 25, 1–47 (1969). 4390734 10.1016/s0022-5193(69)80016-0

[19] J. Jiang, D. Kosman, Y. T. Ip, M. Levine, The dorsal morphogen gradient regulates the mesoderm determinant twist in early Drosophila embryos. Genes Dev. 5, 1881–1891 (1991). 1655572 10.1101/gad.5.10.1881

[20] D. S. Parker, M. A. White, A. I. Ramos, B. A. Cohen, S. Barolo, The cis-regulatory logic of Hedgehog gradient responses: Key roles for gli binding affinity, competition, and cooperativity. Sci. Signal. 4, ra38 (2011). 21653228 10.1126/scisignal.2002077 PMC3152249

[21] K. F. Mayer, H. Schoof, A. Haecker, M. Lenhard, G. Jürgens, T. Laux, Role of WUSCHEL in regulating stem cell fate in the Arabidopsis shoot meristem. Cell 95, 805–815 (1998). 9865698 10.1016/s0092-8674(00)81703-1

[22] H. Schoof, M. Lenhard, A. Haecker, K. F. X. Mayer, G. Jürgens, T. Laux, The stem cell population of Arabidopsis shoot meristems is maintained by a regulatory loop between the CLAVATA and WUSCHEL genes. Cell 100, 635–644 (2000). 10761929 10.1016/s0092-8674(00)80700-x

[23] R. K. Yadav, M. Perales, J. Gruel, T. Girke, H. Jönsson, G. V. Reddy, WUSCHEL protein movement mediates stem cell homeostasis in the Arabidopsis shoot apex. Genes Dev. 25, 2025–2030 (2011). 21979915 10.1101/gad.17258511 PMC3197201

[24] M. Perales, K. Rodriguez, S. Snipes, R. K. Yadav, M. Diaz-Mendoza, G. V. Reddy, Threshold-dependent transcriptional discrimination underlies stem cell homeostasis. Proc. Natl. Acad. Sci. U.S.A. 113, E6298–E6306 (2016). 27671653 10.1073/pnas.1607669113 PMC5068294

[25] S. E. Clark, R. W. Williams, E. M. Meyerowitz, The CLAVATA1 gene encodes a putative receptor kinase that controls shoot and floral meristem size in Arabidopsis. Cell 89, 575–585 (1997). 9160749 10.1016/s0092-8674(00)80239-1

[26] U. Brand, J. C. Fletcher, M. Hobe, E. M. Meyerowitz, R. Simon, Dependence of stem cell fate in Arabidopsis on a feedback loop regulated by CLV3 activity. Science 289, 617–619 (2000). 10915624 10.1126/science.289.5479.617

[27] R. K. Yadav, M. Perales, J. Gruel, C. Ohno, M. Heisler, T. Girke, H. Jönsson, G. V. Reddy, Plant stem cell maintenance involves direct transcriptional repression of differentiation program. Mol. Syst. Biol. 9, 654 (2013). 23549482 10.1038/msb.2013.8 PMC3658276

[28] C. Koppermann, “Crystal structure of the WUSCHEL homeodomain,” thesis, Universität zu Köln, Cologne, Germany (2017).

[29] J. Sloan, J. P. Hakenjos, M. Gebert, O. Ermakova, A. Gumiero, G. Stier, K. Wild, I. Sinning, J. U. Lohmann, Structural basis for the complex DNA binding behavior of the plant stem cell regulator WUSCHEL. Nat. Commun. 11, 2223 (2020). 32376862 10.1038/s41467-020-16024-y PMC7203112

[30] J. Reinitz, S. Hou, D. H. Sharp, Transcriptional control in *Drosophila*. Complexus 1, 54–64 (2003).

[31] M. A. Shea, G. K. Ackers, The OR control system of bacteriophage lambda. A physical-chemical model for gene regulation. J. Mol. Biol. 181, 211–230 (1985). 3157005 10.1016/0022-2836(85)90086-5

[32] X. He, M. A. H. Samee, C. Blatti, S. Sinha, Thermodynamics-based models of transcriptional regulation by enhancers: The roles of synergistic activation, cooperative binding and short-range repression. PLOS Comput. Biol. 6, e1000935 (2010). 20862354 10.1371/journal.pcbi.1000935 PMC2940721

[33] D. Chu, N. R. Zabet, B. Mitavskiy, Models of transcription factor binding: Sensitivity of activation functions to model assumptions. J. Theor. Biol. 257, 419–429 (2009). 19121637 10.1016/j.jtbi.2008.11.026

[34] M. S. Sherman, B. A. Cohen, Thermodynamic state ensemble models of cis-regulation. PLOS Comput. Biol. 8, e1002407 (2012). 22479169 10.1371/journal.pcbi.1002407 PMC3315449

[35] D. T. Gillespie, A general method for numerically simulating the stochastic time evolution of coupled chemical reactions. J. Comput. Phys. 22, 403–434 (1976).

[36] J. Swift, G. M. Coruzzi, A matter of time—How transient transcription factor interactions create dynamic gene regulatory networks. Biochim. Biophys. Acta Gene Regul. Mech. 1860, 75–83 (2017). 27546191 10.1016/j.bbagrm.2016.08.007 PMC5203810

[37] T. O’Brien, J. T. Lis, Rapid changes in Drosophila transcription after an instantaneous heat shock. Mol. Cell. Biol. 13, 3456–3463 (1993). 8497261 10.1128/mcb.13.6.3456-3463.1993 PMC359814

[38] G. K. Ackers, A. D. Johnson, M. A. Shea, Quantitative model for gene regulation by lambda phage repressor. Proc. Natl. Acad. Sci. U.S.A. 79, 1129–1133 (1982). 6461856 10.1073/pnas.79.4.1129 PMC345914

[39] H. G. Garcia, R. Phillips, Quantitative dissection of the simple repression input-output function. Proc. Natl. Acad. Sci. U.S.A. 108, 12173–12178 (2011). 21730194 10.1073/pnas.1015616108 PMC3141941

[40] J. Gertz, E. D. Siggia, B. A. Cohen, Analysis of combinatorial *cis*-regulation in synthetic and genomic promoters. Nature 457, 215–218 (2009). 19029883 10.1038/nature07521 PMC2677908

[41] E. Segal, T. Raveh-Sadka, M. Schroeder, U. Unnerstall, U. Gaul, Predicting expression patterns from regulatory sequence in *Drosophila* segmentation. Nature 451, 535–540 (2008). 18172436 10.1038/nature06496

[42] R. P. Zinzen, C. Girardot, J. Gagneur, M. Braun, E. E. M. Furlong, Combinatorial binding predicts spatio-temporal *cis*-regulatory activity. Nature 462, 65–70 (2009). 19890324 10.1038/nature08531

[43] R. Amit, H. G. Garcia, R. Phillips, S. E. Fraser, Building enhancers from the ground up: A synthetic biology approach. Cell 146, 105–118 (2011). 21729783 10.1016/j.cell.2011.06.024 PMC3155781

[44] E. Davidson, *The Regulatory Genome* (Academic Press, 2006).

[45] H. G. Garcia, A. Sanchez, J. Q. Boedicker, M. Osborne, J. Gelles, J. Kondev, R. Phillips, Operator sequence alters gene expression independently of transcription factor occupancy in bacteria. Cell Rep. 2, 150–161 (2012). 22840405 10.1016/j.celrep.2012.06.004 PMC3616187

[46] C. R. Lickwar, F. Mueller, S. E. Hanlon, J. G. McNally, J. D. Lieb, Genome-wide protein–DNA binding dynamics suggest a molecular clutch for transcription factor function. Nature 484, 251–255 (2012). 22498630 10.1038/nature10985 PMC3341663

[47] J. R. Lipford, G. T. Smith, Y. Chi, R. J. Deshaies, A putative stimulatory role for activator turnover in gene expression. Nature 438, 113–116 (2005). 16267558 10.1038/nature04098

[48] S. H. Spoel, Z. Mou, Y. Tada, N. W. Spivey, P. Genschik, X. Dong, Proteasome-mediated turnover of the transcription coactivator NPR1 plays dual roles in regulating plant immunity. Cell 137, 860–872 (2009). 19490895 10.1016/j.cell.2009.03.038 PMC2704463

[49] F. Geng, S. Wenzel, W. P. Tansey, Ubiquitin and proteasomes in transcription. Annu. Rev. Biochem. 81, 177–201 (2012). 22404630 10.1146/annurev-biochem-052110-120012 PMC3637986

[50] A. Sundqvist, J. Ericsson, Transcription-dependent degradation controls the stability of the SREBP family of transcription factors. Proc. Natl. Acad. Sci. U.S.A. 100, 13833–13838 (2003). 14615581 10.1073/pnas.2335135100 PMC283507

[51] A. Plong, K. Rodriguez, M. Alber, W. Chen, G. V. Reddy, CLAVATA3 mediated simultaneous control of transcriptional and post-translational processes provides robustness to the WUSCHEL gradient. Nat. Commun. 12, 6361 (2021). 34737298 10.1038/s41467-021-26586-0 PMC8569176

[52] S. A. Snipes, K. Rodriguez, A. E. DeVries, K. N. Miyawaki, M. Perales, M. Xie, G. V. Reddy, Cytokinin stabilizes WUSCHEL by acting on the protein domains required for nuclear enrichment and transcription. PLOS Genet. 14, e1007351 (2018). 29659567 10.1371/journal.pgen.1007351 PMC5919686

[53] K. Rodriguez, M. Perales, S. Snipes, R. K. Yadav, M. Diaz-Mendoza, G. V. Reddy, DNA-dependent homodimerization, sub-cellular partitioning, and protein destabilization control WUSCHEL levels and spatial patterning. Proc. Natl. Acad. Sci. U.S.A. 113, E6307–E6315 (2016). 27671631 10.1073/pnas.1607673113 PMC5068338

[54] R. Sayal, J. M. Dresch, I. Pushel, B. R. Taylor, D. N. Arnosti, Quantitative perturbation-based analysis of gene expression predicts enhancer activity in early Drosophila embryo. Elife 5, e08445 (2016). 27152947 10.7554/eLife.08445 PMC4859806

[55] Y. Zhou, X. Liu, E. M. Engstrom, Z. L. Nimchuk, J. L. Pruneda-Paz, P. T. Tarr, A. Yan, S. A. Kay, E. M. Meyerowitz, Control of plant stem cell function by conserved interacting transcriptional regulators. Nature 517, 377–380 (2015). 25363783 10.1038/nature13853 PMC4297503

[56] Y. H. Su, C. Zhou, Y. J. Li, Y. Yu, L. P. Tang, W. J. Zhang, W. J. Yao, R. Huang, T. Laux, X. S. Zhang, Integration of pluripotency pathways regulates stem cell maintenance in the *Arabidopsis* shoot meristem. Proc. Natl. Acad. Sci. U.S.A. 117, 22561–22571 (2020). 32839309 10.1073/pnas.2015248117 PMC7486707

[57] M. Kieffer, Y. Stern, H. Cook, E. Clerici, C. Maulbetsch, T. Laux, B. Davies, Analysis of the transcription factor WUSCHEL and its functional homologue in Antirrhinum reveals a potential mechanism for their roles in meristem maintenance. Plant Cell 18, 560–573 (2006). 16461579 10.1105/tpc.105.039107 PMC1383633

[58] B. Senay-Aras, W. Chen. Stochastic cis-elements binding model (2022); https://zenodo.org/badge/latestdoi/349283086.

[59] A. Do, MeristemBasic_p (2022); https://zenodo.org/badge/latestdoi/501822165.

[60] A. Do, BasicCisElementAnalyzer (2022); https://zenodo.org/badge/latestdoi/498280124.

[61] M. R. Roussel, R. Zhu, Validation of an algorithm for delay stochastic simulation of transcription and translation in prokaryotic gene expression. Phys. Biol. 3, 274–284 (2006). 17200603 10.1088/1478-3975/3/4/005

[62] E. Azpeitia, A. Wagner, Short residence times of DNA-bound transcription factors can reduce gene expression noise and increase the transmission of information in a gene regulation system. Front. Mol. Biosci. 7, 67 (2020). 32411721 10.3389/fmolb.2020.00067 PMC7198700

[63] P. S. Gutierrez, D. Monteoliva, L. Diambra, Cooperative binding of transcription factors promotes bimodal gene expression response. PLOS ONE 7, e44812 (2012). 22984566 10.1371/journal.pone.0044812 PMC3440358

[64] U. Brand, M. Grünewald, M. Hobe, R. Simon, Regulation of CLV3 expression by two homeobox genes in Arabidopsis. Plant Physiol. 129, 565–575 (2002). 12068101 10.1104/pp.001867 PMC161677

