# Supplementary material for: Concentration-dependent transcriptional switching through a collective action of cis-elements
Source: Sci Adv. 2022 Aug 10;8(31):eabo6157. doi: 10.1126/sciadv.abo6157 (PMC9365274; doi:10.1126/sciadv.abo6157)
Supplement: Supplementary file 1 — Supplementary Text Figs. S1 to S11 Tables S1 to S5 References [file sciadv.abo6157_sm.pdf]

Science Advances
AAAS

Supplementary Materials for

Concentration-dependent transcriptional switching through a collective action of cis-elements

Kevin Rodriguez *et al.*

Corresponding author: G. Venugopala Reddy, venug@ucr.edu; Weitao Chen, weitaoc@ucr.edu

Sci. Adv. **8**, eabo6157 (2022)
DOI: 10.1126/sciadv.abo6157

The PDF file includes:

Supplementary Text
Figs. S1 to S11
Tables S1 to S5
Legends for tables S6 and S7
Legend for data S1
References

Other Supplementary Material for this manuscript includes the following:

Tables S6 and S7
Data S1

Supplementary Text

Plant growth conditions and genotypes.

All plants were grown at 25°C under continuous light. All transgenic plants were generated in the Landsberg *erecta* background. The wild-type p*CLV3::H2B-mYFP* reporter has been described earlier (*24*). Various cis-element mutant p*CLV3::H2B-mYFP* reporters described were generated through PCR mutagenesis by using appropriate primers listed in Table S4. The sequence of the *pCLV3::H2B-mYFP* and *pCLV3::CLV3genomics* are included in Data S1, while the mutant CRM excerpts are included in Table S5. The p*35S::eGFP-WUS-GR* transgenic plants described in the earlier study (*53*) were crossed to the wild-type p*CLV3::H2B-mYFP*, p*CLV3 (DM)::H2B-mYFP* and p*CLV3 (QM)::H2B-mYFP* reporters (*24*). The progeny was exposed to mock or 10μM Dexamethasone (Dex) for 24 hours and imaged as described below.

Sample preparation and confocal microscopy.

The images were acquired from three-week-old plants. All surrounding older flowers were removed carefully. The excised stem containing the shoot apex was transplanted into a plastic imaging box containing a 1 cm thick layer of 1.5 % agarose. The stem was stabilized by pouring additional amounts of molten agarose, then submerged in deionized water and further processed under a stereomicroscope. The remaining older floral buds covering the SAM were further trimmed with tweezers to expose the SAM. The water was discarded and a droplet of 3% FM-4-64 dissolved in deionized water containing 0.016% silwet-77 was applied to each SAM. After 10 minutes of FM4-64 staining, the plants were submerged in deionized water and imaged by using the Zeiss880 confocal microscope.

Image quantification and analysis

The p*CLV3::H2B-mYFP* reporter expression and *pWUS::eGFP-WUS* nuclear protein accumulation were quantified from four independent SAMs. The mean nuclear fluorescence signal from the ten central most cells in each cell layer were manually selected using a circle tool within the ZEN 2.3 blue edition software. To evaluate the statistical significance, two-tailed t-tests were applied comparing wild-type *CLV3* reporter levels in each cell layer to various mutant reporters described. The quantification of SAM height was carried out as described in (*24*). Two samples on each independent line, for a minimum of nine independent transgenic lines, for each WUS binding cis-element promoter were analyzed. The height was determined from the junction of the 5th primordia to the SAM apex. The carpel number was performed on two samples on each independent line, for a minimum of 5 independent transgenic lines. The multiple comparison was performed by one-way ANOVA followed by Tukey's HSD test using R (v3.6.1) package.

Gel shift assays - EMSA

The purification of wild-type WUS protein (amino acids 1-292) was carried out as described previously in (*24*). His-WUS protein, cloned into pET28*α* plasmid (Novagen), was expressed in BL21 cells. WUS protein was purified from the soluble lysate using a His-tag protocol (Ni-NTA His-Bind Resins; Novagen) and dialyzed in 20 mM Hepes at pH 7.8 and 100 mM KCl. The EMSA was carried out as described in an earlier study (*23, 24*). Oligonucleotides were radiolabeled with (Ɣ-32P) ATP by T4 polynucleotide kinase (NEB) and annealed with complementary oligonucleotides to make double stranded DNA. The protein-DNA binding reaction was performed in a 20 μL reaction mixture: 10fmol probe, 1x binding buffer (20mM HEPES-KOH at pH7.8, 100mM KCl, 1mMEDTA, 0.1% BSA, 200 ng DNA salmon sperm, and 10% glycerol) and His-WUS protein. After a 20 min incubation at RT, samples were loaded into a 6% native polyacrylamide gel. Electrophoresis was performed at 10V/cm for 90 min in 0.5x Tris-borate buffer. Gels were autoradiographed using phospho imaging and Typhon system. The single cis-element probes were same as those used in earlier study (*24*). The sequences of new

oligonucleotide probes containing two cis-elements, the corresponding mutant forms, and probes with altered intervening distance have been listed in Table S2.

BiFC analysis

For the BiFC analyses, the N-terminal fragment of eGFP (NeGFP) from nucleotides 1-465 (AA 1-155), and the C-terminal fragment of eGFP from nucleotides 466-723 (AA 156-241) were generated through an inverse PCR using 5' Phosphorylated oligos in (Table S4) on the PCR4 cloning vector with eGFP-WUS described in (*23*). For generating stable transgenic lines, these constructs were introduced into the *WUS promoter* described in earlier study (*23*). Fifteen independent transgenic plants for each construct were isolated and crossed to each other. The F1 progeny were used for visualizing BiFC signal in SAM tissue.

Description of stochastic single-cell model

In the stochastic single-cell model, the WUS concentration-dependent activation and repression of *CLV3* transcription is modeled as follows. WUS can bind as a monomer or dimer to each of the five cis-elements at different affinities. In particular, it is assumed that only monomers can recruit Pol II for transcription of *CLV3* and one molecule of *CLV3 mRNA* is generated for each Pol II recruitment. The total amount of *CLV3 mRNA* produced is quantified. Specifically, the following five possible stochastic events are considered, where S represents a binding site, P denotes one WUS molecule, SP denotes the WUS monomer bound cis-element, SP_2 denotes a WUS dimer bound cis-element, TSS is the transcription starting site, $Pol\ II$ is RNA Polymerase and $mRNA$ denotes *CLV3 mRNA*:

$S + P \xrightarrow{k_{on}^M} SP$ binding with one WUS molecule (WUS monomer) [1]

$SP \xrightarrow{k_{off}^M} S + P$ unbinding of WUS monomer creates unoccupied cis-element [2]

$SP + P \xrightarrow{k_{on}^D} SP_2$ binding of the second WUS molecule to the WUS monomer (WUS dimer) [3]

$SP_2 \xrightarrow{k_{off}^D} SP + P$ unbinding of the second WUS molecule generates WUS monomer [4]

$TSS + SP + Pol\ II \xrightarrow{k_p} TSS(t_1) + SP + Pol\ II(t_2) + mRNA(t_2)$
Recruitment of Pol II by a WUS monomer bound to a cis-element to produce *CLV3* mRNA [5]

In EQ (1) and (2), a single WUS molecule binds an empty cis-element at a rate k_{on}^M $(\mu M^{-1}s^{-1})$ and the monomer dissociates at a rate $k_{off}^M(s^{-1})$. Similarly in EQ (3) and (4), a second WUS molecule binds a WUS monomer at a rate k_{on}^D $(\mu M^{-1}s^{-1})$ and a dimer dissociates at a rate $k_{of}^D(s^{-1})$. EQ.5 represents the recruitment of Pol II by a WUS monomer bound to a cis-element, SP, at TSS for *CLV3* mRNA transcription. Pol II molecules are recruited to Transcription Start Site (TSS) at a rate $k_p(s^{-1})$ by any WUS monomer present in CRM. There is a time delay for the release of TSS, Pol II and mRNA. TSS is released with a delay t_1, Pol II and mRNA is released with a delay t_2. We apply the Gillespie algorithm, developed originally to simulate discrete interactions in chemical reaction systems, through the use of Monte Carlo method in determining time step sizes and occurring events. By applying this algorithm, possible events and their associated probabilities are updated in each iteration according to the current status of the cis-elements. In particular, the delayed stochastic simulation algorithm (SSA) (*61*) was applied based on the estimated 4 sec time delay (denoted by $t_1 = t_{delay}$ in the algorithm), involved in the recruitment of successive Pol II molecules to the transcription start site. Assuming $k_{on}^M = k_{on}^D = k_{on}$ for all sites, the association and dissociation probabilities of monomers are given by (*62*):

$$p_a = \frac{k_{on}}{VN_A} N_P N_S \quad [6]$$

$$p_d = k_{off} N_{SP} \quad [7]$$

where V is the reaction volume, N_A is the Avogadro's number, N_P, N_S and N_{SP} are the number of WUS molecules, cis-elements and WUS monomer bound cis-elements respectively. We consider WUS binding and unbinding events to each cis-element as a separate event and therefore $N_s = N_{SP} = 1$ (or 0 depending on the state of cis-elements in the CRM). Also $\frac{N_P}{VN_A} = [WUS]$ where $[WUS]$ is the concentration of WUS proteins. In the thermodynamic equilibrium, we have $K_d = \frac{k_{off}}{k_{on}}$**,** where K_d is the affinity of the cis-element and it is determined by EMSA (*24*). Combining these we obtain:

$$p_a = k_{on}[WUS] \quad [8]$$

$$p_d = k_{off} = k_{on} K_d \quad [9]$$

Considering k_{on} as a free parameter, we can conclude that binding propensities depend on WUS concentration and unbinding propensities of WUS monomers depend on affinities of cis-elements. WUS dimers are also modeled similarly by estimating K_d for dimerization according to EMSA data.

Additional assumptions

- WUS concentration is assumed to be constant in time since each binding or unbinding event is not expected to change the overall WUS concentration in the nucleus.
- Dimers are formed in two steps, i.e., first one WUS molecule binds as a monomer and then it becomes dimer if the second WUS molecule binds, as shown in EMSA experiments (*24*).
- If a WUS monomer does not cooperate with other WUS monomers to stabilize each other, it dissociates upon reaching the "residence time limit (RTL)" as explained in the main text. This dissociation of WUS monomers is not directly related to affinities of the cis-elements when only a single cis-element is functional.
- WUS monomers bound to the cis-elements recruit Pol II for transcribing *CLV3* and each Pol II recruitment generates one *CLV3* mRNA. Successive recruitment of Pol II to the transcription start site (TSS) is assumed to happen with an estimated 4 sec time delay. The time delay ($t_1 = t_{delay}$) is based on an 80 bp footprint of Pol II containing protein complex and mRNA elongation rate, which is estimated to be 1.2 kb/min (*37*) $[80bp \times (60\ sec/1200\ bp) = 4\ sec]$. Pol II and *CLV3* mRNA are released with a delay of t_2 seconds upon completion of the transcription, but we assume Pol II is abundant in the system so the total number is unaffected. Since we track the total number of *CLV3* mRNA generated at steady-state, we neglect the second time delay t_2.
- Right after the recruitment of one Pol II by a WUS monomer, the WUS monomer can unbind the cis-element or a second WUS monomer can bind to generate WUS dimer on the same cis-element without affecting transcribing by Pol II.
- Degradation of *CLV3* mRNA is neglected. We tested single cis-element simulations with different degradation values. When degradation rate is high compared to the binding and unbinding rates of WUS molecules, the simulations failed to capture the single cis-element *CLV3* expression behavior observed in the experiments. When degradation is much smaller than binding and unbinding rates of WUS molecules, the simulations could capture the experimental data qualitatively and moreover, the degradation level did not affect the *CLV3* expression pattern qualitatively. Therefore, for simplicity we neglected the degradation of *CLV3* mRNA.

At any given time, the next event can be one of the five stochastic events described in Eq. (1-5), the release of Pol II from TSS (at the end of $t_{delay} = 4s$), or the release of a WUS monomer

with no cooperativity upon reaching the "residence time limit". The remaining time for the release of Pol II from the TSS, the minimum of the remaining times for the release of the monomers bound to cis-elements upon reaching the residence time limit and the waiting period for the next stochastic event are denoted by t_{rem}^1, t_{rem}^2 and τ respectively.

Algorithm

1. Set $t = 0$, the amount of *CLV3* mRNA is zero and TSS and *CLV3* CRM are unoccupied.
2. Propensity functions are calculated for each possible event based on current binding status, denoted by $a_i(t)$ for each event. The sum is denoted by $a_0(t) = \sum_{i=1}^{N} a_i(t)$.
3. Two random numbers r_1 and r_2 are generated from a standard uniform distribution.
4. The waiting time for the next event is calculated as $\tau = -ln(r_1)/a_0$; the next event with the index j satisfying $\sum_{i=1}^{j-1} a_i(t) \leq a_0 r_2 < \sum_{i=1}^{j} a_i(t)$ is chosen.
5. Compare τ with t_{rem}^1 and t_{rem}^2. Apply one of a, b or c below:
    a. If *CLV3* CRM is bound with a WUS monomer with no cooperativity and (($t_{rem}^1 < min(\tau, t_{rem}^2)$) or ($t_{rem}^1 < \tau$ and TSS is unoccupied)): Update the time as $t \leftarrow t + t_{rem}^1$ and update *CLV3* CRM by unbinding the WUS monomer, set $t_{rem}^1 = 0$ and if TSS is occupied, update $t_{rem}^2 = t_{rem}^2 - t_{rem}^1$.
    b. If Pol II is bound to TSS and ($t_{rem}^2 < min(\tau, t_{rem}^1)$) or ($t_{rem}^2 < \tau$ and *CLV3* CRM is not bound to a WUS monomer with no cooperativity)): Update the time as $t \leftarrow t + t_{rem}^2$, increase the amount of *CLV3* mRNA by 1, set $t_{rem}^2 = 0$ and set TSS to be unoccupied. If at least one WUS monomer with zero cooperativity is bound, update $t_{rem}^1 = t_{rem}^1 - t_{rem}^2$.
    c. If ($\tau < min(t_{rem}^1, t_{rem}^2)$) or (TSS is unoccupied and $\tau < t_{rem}^1$) or (*CLV3* CRM is not bound to a WUS monomer with no cooperativity and $\tau < t_{rem}^2$) or (TSS is unoccupied and *CLV3* CRM is not bound to a WUS monomer with no cooperativity): Update the time as $t \leftarrow t + \tau$ and update the current state of the *CLV3* CRM or TSS according to the selected event j. If TSS is occupied, update $t_{rem}^2 = t_{rem}^2 - \tau$. If at least one WUS monomer with zero cooperativity is bound, update $t_{rem}^1 = t_{rem}^1 - \tau$. If the selected event is Pol II recruitment, set $t_{rem}^2 = t_{delay}$. If the selected event is a WUS monomer binding without cooperativity, set $t_{rem}^1 = RTL$.
6. If $t < t_{final}$, go to Step 2, otherwise stop.

For each stochastic simulation, the activation of *CLV3* is obtained by calculating the total amount of mRNA accumulated during the simulation period. The mean value from a sufficiently large number of independent simulations (*41*) was generated and compared with the experimentally quantified *CLV3* expression measured by fluorescence quantification of *pCLV3::H2B-mYFP* (Fig. 2). In Fig. 4, 6A-E and Fig. S5, $t_{final} = 1.6 \times 10^6$ in the simulations, and in Fig. S9, $t_{final} = 1.6 \times 10^5$.

Parameters

The K_d values for events EQ.1 and EQ.2, which are binding and unbinding events of a WUS monomer to different cis-elements, are represented by $K_d = k_{off}^M / k_{on}^M$ and they are determined by the EMSA experiments (*24*). With these K_d values (Table S1), first we simulated WUS monomer binding and unbinding and compared the simulation results with the quantitative data from EMSA experiments with WUS that lacked the C-terminal homodimerization domain. In these simulations, we chose k_{on} values between 0.1 and 1 which fit well to the experimental data (Fig. S4A). Next, we simulated monomer and dimer binding and unbinding similar to the EMSA experiments with WUS that had the C-terminal homodimerization domain. Since the quantitative

data for WUS dimerization (K_d values) is not available, we matched EMSA data qualitatively as follows. We simply considered ${K_d}^d$, the dimerization K_d, the same as monomer K_d and we chose $k_{on}^M = k_{on}^D = 0.1$. We tested this assumption by simulating the single cis-element EMSA experiments with WUS that had the C-terminal homodimerization domain. Since the quantification of the proportions of monomers and dimers in these experiments is not possible, we compared our simulations to the EMSA experiments qualitatively. For the cis-elements 970i, 970M4i, 997i and 1007i, the assumption $K_d^d = K_d$ could generate results similar to the data qualitatively, so we proceeded with this assumption for these cis-elements. But for the low affinity cis-elements 950i and 1060i, the assumption that $K_d^d = 0.5K_d$ gave better results. In summary, we defined $k_{off}^M = K_d \cdot k_{on}$ and $k_{off}^D = K_d^d \cdot k_{on}$ with $k_{on} = 0.1$ (Fig. S4B). In EQ5, k_p is a free parameter that was tested to ascertain how varying k_{on} and k_p affects intrinsic activation of *CLV3* in the case of highest (970M4i) and lowest affinity (1060i) cis-elements (Fig. S9). The total amount of transcriptional output increased as k_p increased from 0.2 to 100 for fixed k_{on}. To calibrate k_{on} and k_p values, we simulated our model for highest affinity single cis-element 970M4i and lowest affinity single cis-element 1060i, and calculated the ratio of the experimental *CLV3* reporter values for 970M4i and 1060i in different cell layers. For small k_{on} values like $k_{on}=1$ or smaller, we see a huge difference between simulations and experimental data. For larger values such as $k_{on}=10$ and $k_p=10$, the ratio of expressions of 970M4i and 1060i gets closer to experimental measurements (Fig. S11A). However, for multiple cis-element behavior with $k_{on}=10$ and $k_p=10$ (WT, 970M, 950M and DM [970M-997M]), we observed high levels of expression at low WUS concentrations for all mutants including 970M and DM, similar to the wild-type, which is contrary to the experiments. We also tried simulations under different cooperativity assumptions and levels and obtained similar results that didn't match the experiments (Fig. S11B-D). Therefore, although it is possible to generate the single cis-element behavior using larger values of k_{on} and k_p, the multiple cis-element behavior could not be generated. In contrast, using smaller values of k_{on} and k_p coupled with the monomer residence time limit, the model was able to generate both single and multiple cis-element behaviors consistent with experiments. Therefore, $k_p=0.2$ and $k_{on}=0.1$ are used in all simulations of the single cell model.

The WUS levels in different cell layers in the single cell model were chosen to satisfy the experimental quantification where WUS concentration decreases by ~1.5 fold from L2 to L1 and ~2 fold from L3 to L2 (*51*). The chosen WUS levels were able to generate the experimentally observed *CLV3* expression.

Cooperativity

The free energy of binding of a WUS molecule to a cis-element can also be affected by the interaction between WUS bound to other cis-elements in the CRM. This interaction between WUS molecules might increase the binding propensity of the new WUS molecules or stabilize the bound ones, e.g. decrease the unbinding propensity of the bound WUS molecules (*63*), which was modeled as cooperativity. We first considered equal cooperativity between every two cis-elements in the model, which couldn't generate consistent results as the experimental data (Fig. 6C). Cooperativity as a function of the distance between binding sites, d, was studied before (*54*). To model the strength of the interaction between WUS molecules bound to different cis-elements, we defined a function $f(d)$ which decreases as d increases. To model the increase in the binding propensity due to cooperativity we multiplied the original propensity with $f(d)$ and to model the decrease in unbinding propensity we divided that with $f(d)$. A linear function did not give rise to biologically consistent behavior, therefore we considered the following nonlinear function: $f(d) = -a_d \, arctan\,(a_c(d - b_d)) + 1.5708\, a_d + 1$ to reduce the difference between wild-type and 950M on *CLV3* expression pattern. If $a_i(t) = k_{on}WUS$ is the propensity function for binding

of a TF to a cis-element when there is no interaction, the binding propensity becomes $a_i(t) = k_{on}WUS(\Pi_{j=1}^{n-1} f(d_j))$ where d_j is the distance between any two cis-elements and n is the number of functional cis-elements that are bound with WUS monomers (or dimers). We chose this function in order to reproduce the experimental observations for simplicity. The function f constructed in this way satisfies that: if the distance between two cis-elements is less than a threshold value, then f takes some value much greater than 1, representing the cooperativity is strong; if the distance between two cis-elements is greater than another threshold value, then $f(d)$ becomes close or equal to 1, which means a decreased or zero cooperativity; if the distance is between these two threshold values, then $f(d)$ decreases as the distance increases. Similarly, the unbinding propensity of a TF molecule is $a_i(t) = k_{off}$ when there is no interaction between bound molecules and it becomes $a_i(t) = k_{off}/(\Pi_{j=1}^{n-1} f(d_j))$ when there is some interaction. In summary, the cooperativity between cis-elements increases binding probability and also decreases unbinding probability of WUS molecules. The parameters for the cooperativity was determined by comparing the *CLV3* expression obtained in simulations to the experimentally observed *CLV3* expression patterns of various cis-element mutants. The model description of cooperativity used to generate results presented in Fig. 6 is provided below.

Fig. 6A: No cooperativity between cis-elements is considered. Unbinding propensity $a_i(t) = k_{off}$ stays the same.

Fig. 6B, C: WUS dimer cooperativity is included, which is equal between every cis-element independent of the intervening distance. Unbinding propensity becomes $a_i(t) = k_{off}/(a_d + 1)^{n-1}$ where n is the number of functional cis-elements that are bound with dimers and a_d is a constant. $a_d = 2,4,6,8,10$ are tested for the simulation results presented in the Fig 6B.

Fig. 6D: Distance dependent WUS dimer cooperativity is considered. Unbinding propensity becomes $a_i(t) = k_{off}/(\Pi_{j=1}^{n-1} f(d_j))$. $a_d = 40$ and $b_d = 40$ in these simulations.

Fig. 6E: Distance dependent WUS monomer and dimer cooperativity is considered. Binding propensity becomes $a_i(t) = k_{on}WUS(\Pi_{j=1}^{n-1} f(d_j))$ and unbinding propensity becomes $a_i(t) = k_{off}/(\Pi_{j=1}^{n-1} f(d_j))$. The parameters for these simulations are given in Table S2.

Description of 3D cell-based model

We model the SAM by a half dome shape consisting of more than 1000 unit spheres representing individual cells (*23*). The simulated SAM is divided into multiple layers. The first layer of cells represents Layer 1 (L1) and it includes all cells with centers higher than 8.5 units above the center of the base of the SAM. The next layer of cells represents Layer 2 (L2) and includes all cells with centers between 8-8.5 units above the base center of the SAM. Below this the layers are defined as follows: L3: 8-6.5 units, L4: 7 and 6 units, L5: 6 and 5 units, L6: 5 and 4 units, and L7: 4 and 3 units. Production of *CLV3* mRNA is limited to a cylinder shaped spatial domain and the radius of the cylinder is given in Table S3. Since in each cell there exists two copies of CRMs, we consider two independent identical groups of 5 cis-elements in the model. Each simulation is run sufficiently long to achieve the steady-state behavior. The 3D cell-based model follows the same approach as the stochastic single-cell model for individual cells, except the following modeling assumptions:

1. WUS gradient at the tissue level. A gradient of nuclear WUS proteins is chosen to be consistent with the experimental quantifications across different layers and is fixed throughout simulations under different mutant cis-elements conditions. The local concentration of WUS is

then used in the stochastic binding model inside each cell to obtain the *CLV3* expression pattern in the tissue.

2. Stochastic binding dynamics in individual cells. The stochastic single-cell model is applied for each CRM inside individual cells with some minor modifications described as below:

i. For all binding events, the propensity of binding becomes $k_{on}\ WusConc/WusSat$ instead of $k_{on}\ WusConc$ used in the stochastic single-cell model. This is because WUS concentrations used in the cell-based model have a different scale from the one in the single-cell model.

ii. For the monomer cooperativity, it is assumed that 970 plays a dominant role due to its higher affinity. In particular, the propensity function for monomer unbinding events associated with 970 is $k_{on}K_d c_{coop}^{n+n_{970}}$, where n is the number of WUS monomers bound to all other cis-elements and n_{970} is a constant calibrated in the model (Table S3). For all other cis-elements, the propensity function for monomer unbinding events becomes $k_{on}K_d c_{coop}^{n}$, where n is the number of WUS monomers bound to 970 and $c_{coop} = monomerCoopt$ is a parameter calibrated in the model (Table S3, Fig. 9-10).

Regarding the dimer cooperativity, for all five cis-element, the propensity function for dimer unbinding events is $k_{on}K_d c_{coop}^{n}$, where n is the number of WUS dimers bound to neighboring nonempty cis-elements. For 970M4, due to its enhanced affinity, the propensity function for dimer unbinding event is $k_{on}K_d c_{coop}^{n+n_{970}}$, where n is the number of WUS dimers bound to all other cis-elements and $c_{coop} = dimerCoopt$ is a parameter calibrated in the model (Table S3, Fig. 9-10).

The cooperativity modeled in this way is similar to the one used in the single-cell model which has a threshold such that the cooperativity associated with the closest (or adjacent) unoccupied neighbors is much stronger.

3. Parameters. This 3D cell-based model involves multiple parameters which can't be estimated directly from experimental data. In particular, those involved in the stochastic binding dynamics in each cell are calibrated using the stochastic single-cell model and the same values are adopted. Parameters that are involved in the 3D cell-based model only are provided in Table S3.

Bioinformatic analysis of Cis-element clusters in WUSCHEL regulated genes

To search for the occurrence of cis-element clusters in WUS regulated genes, a list of genes that were upregulated and downregulated by WUS (*27*) in the presence of protein synthesis inhibitor Cyclohexamide were considered. The search begins with the main function Arabmotifsearch(). A target file containing the WUS regulated genes, a GFF file containing annotation information, and the TAIR10 sequence are input along with the desired names of the output files. The annotation file is broken into several pieces including chromosome, gene names, and start and end positions.

For each gene target, the function targetScanner() is run, associating the target gene with a chromosome and sequence. The function MotifPrelimScanner() scans the sequence defined from 3000 bp before the gene start (Transcription start site) to 3000 bp after the gene end (Transcription stop site) for TAAT/ATTA cis-elements. The cis-elements list is fed into ClusterScanner() to detect cis-element clusters/CRMs which are defined as strings of at least 4 cis-elements which are within 50 bp of the previous one.

Each CRM is then scanned for complex cis-elements in Complexcorescanner() which is defined as a string of cis-elements where each cis-element is 4bp or less from the previous one. Then complexcore score; which is the length of complex cis-elements of the CRMs are summed up for

each cis-element cluster. Motifscorer() elements calculates phasing score which is defined by how well consecutive cis-elements adhere to a 10.5x bp spacing relationship that allows binding of WUS molecules on the same side of the DNA. To normalize the phasing score, the phasing per base was calculated; where the phasing score is divided by the number of bp of the CRM.

These results are arranged by gene and by cluster into columns and printed out by Motifwriter(). This data is provided in Tables S6 and S7 as an excel and BED format. The data and viewing instructions are provided in the specified link.
[https://github.com/Ado012/BasicCisElementAnalyzer]

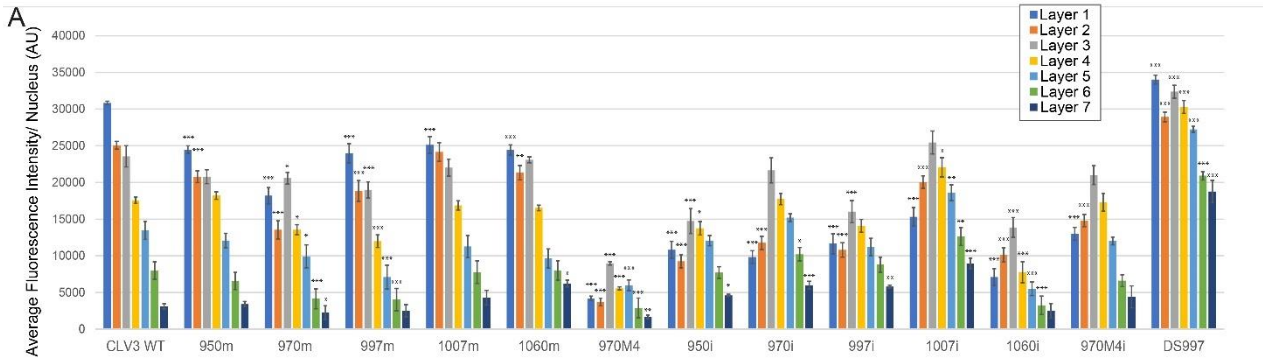

Fig. S1. Quantification of *pCLV3::H2B-mYFP* fluorescence levels in wild-type and various cis-element mutants.

Average fluorescence levels (mean ±S.E.) of H2B-mYFP in different cell layers of wild-type and various *pCLV3::H2b-mYFP* promoter variants indicated below each group. Mutation in single cis-elements (950M, 970M, 997M, 1007M, 1060M and 970M4), mutations in four of the five cis-element mutants [quadruple mutants-950i, 970i, 997i, 1007i, 1060i and 970M4i], and variant with doubled intervening distance between 970-997 and 997-1007 (DS-997). The error bars represent the standard error (n=4 biological replicates in all cases). AU, arbitrary unit. * $p<0.05$, ** $p<0.01$, *** $p<0.001$

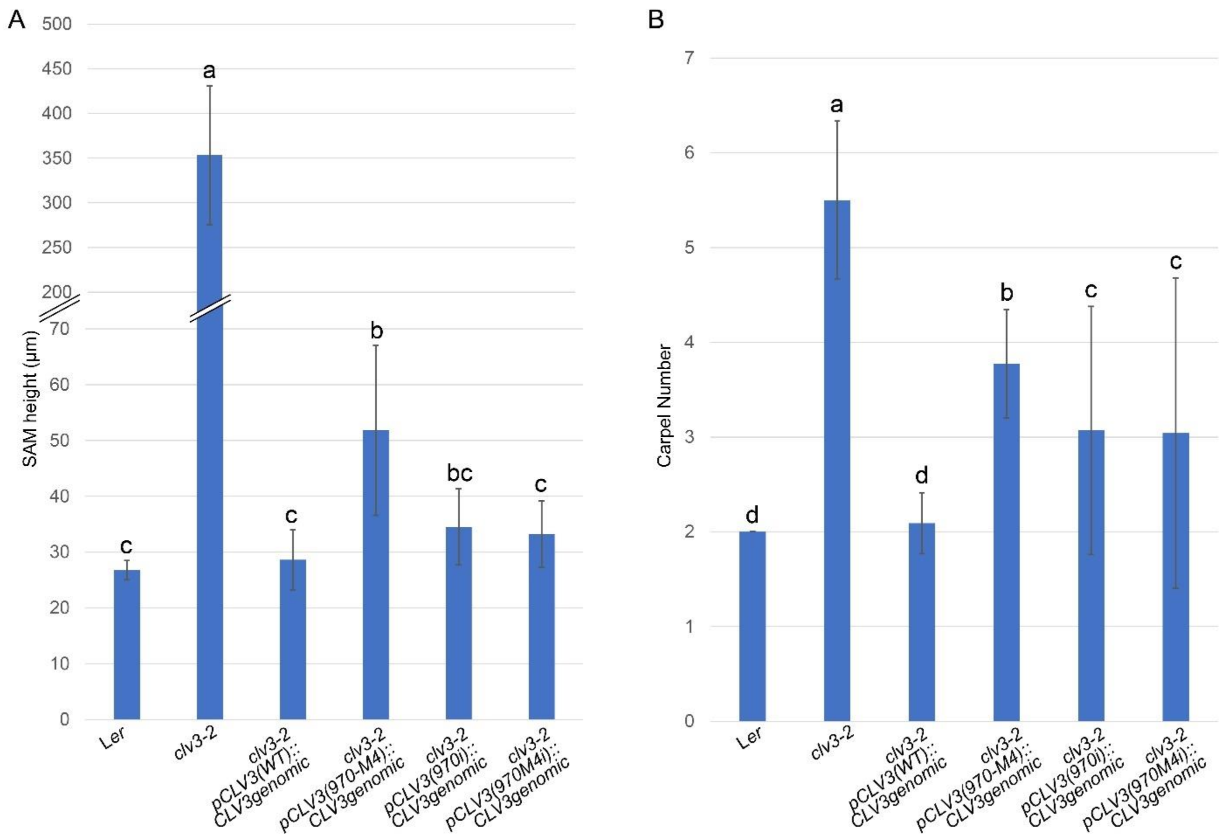

Fig. S2. Number and affinity of *CLV3* cis-elements determine the SAM and floral meristem complementation of *clv3* null mutants. (A) Average SAM height (mean ±S.D.) of wild-type, *clv3-2* mutants and various cis-element mutants of *pCLV3::CLV3* genomic constructs rescuing *clv3-2* mutants. (n=20, 2 plants from each of the ten independent transformed lines were considered). (B) Average number of carpels (mean ±S.D.) of the same genotypes described in (A). (n=10, 2 plants from each of a minimum of 5 independent transformed lines were considered). The different letter indicate the statistical difference between lines (*P<0.05*) as determined by Tukey's Honest Significant Difference (HSD) tests.

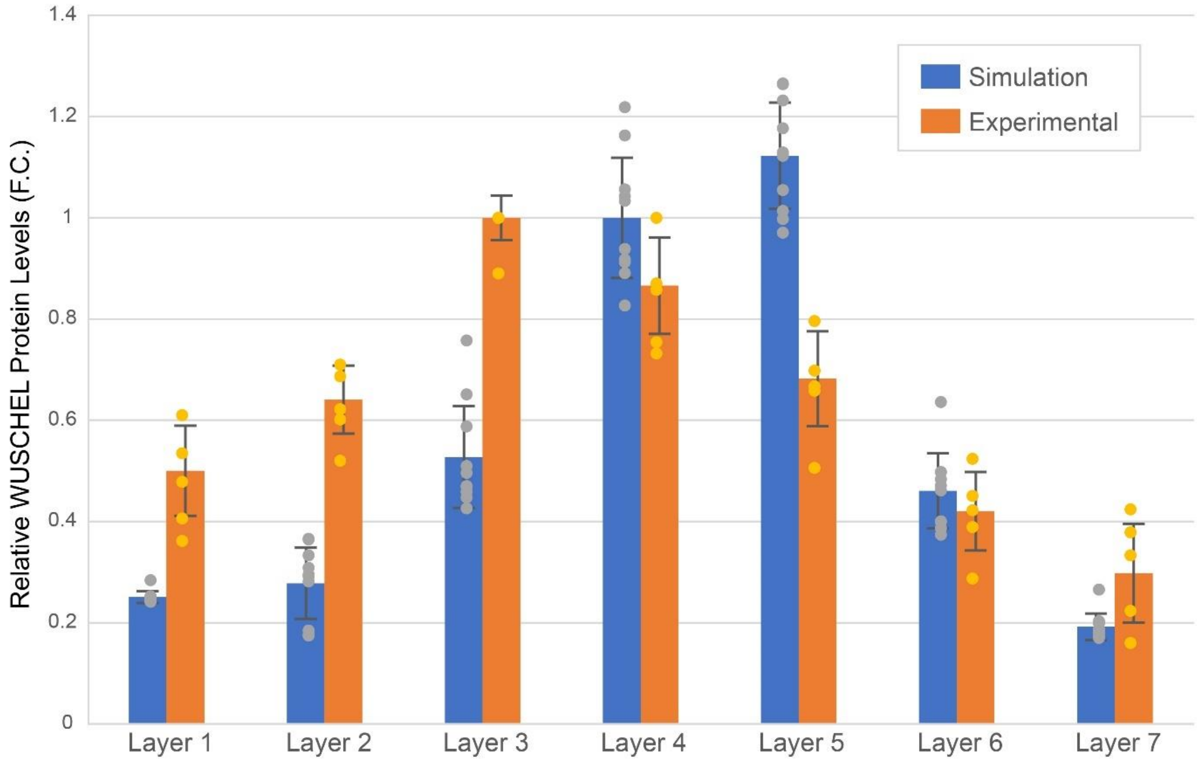

Fig. S3. Quantified WUS protein levels in different cell layers.

For the WUS levels in simulations, the ten cells with the highest WUS levels were selected for each cell layer. WUS levels for each cell were normalized to the cell with the highest WUS nuclear level (mean ±S.D.). For experimental quantification (orange), the average nuclear fluorescence levels for ten cells per layer of pWUS::eGFP-WUS were quantified from five independent SAMs (mean ±S.D.). WUS concentration in each cell layer was normalized by the mean of the layer with the highest WUS level. F.C., fold change.

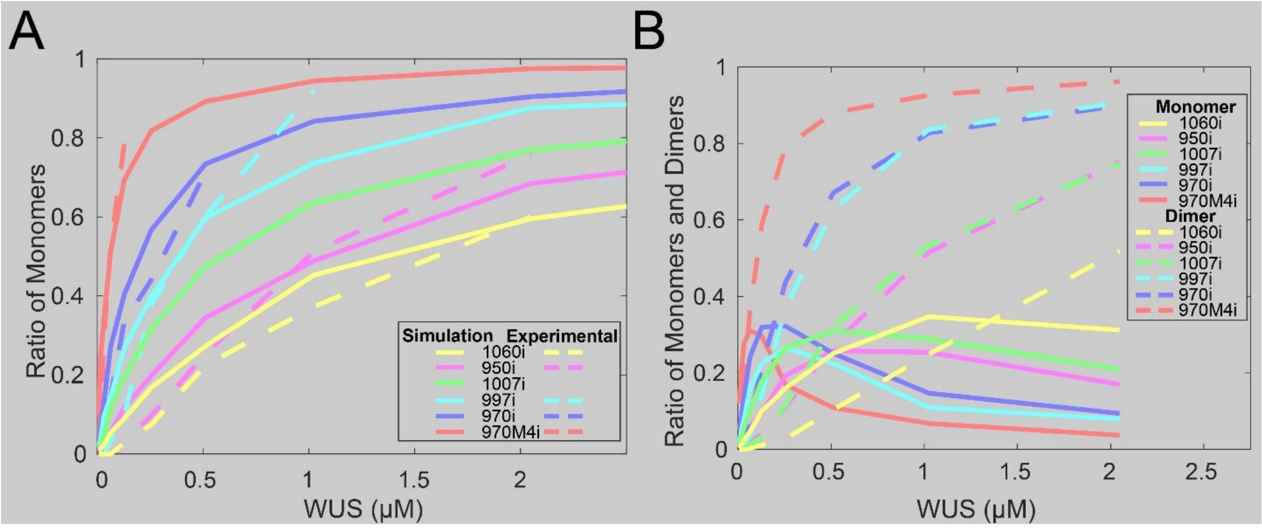

Fig. S4. Simulated WUS monomers and dimers at increasing WUS concentrations for various *CLV3* promoter variants.

(**A**) WUS monomers compared to the experimentally quantified data from EMSA. Solid lines represent simulation results and dashed lines represent values from EMSA data. (**B**) Simulated ratios of WUS monomers and dimers with increasing WUS concentrations. Solid lines represent WUS monomer species and dashed lines represent WUS dimers. The colored lines represent different *CLV3* cis-element mutants.

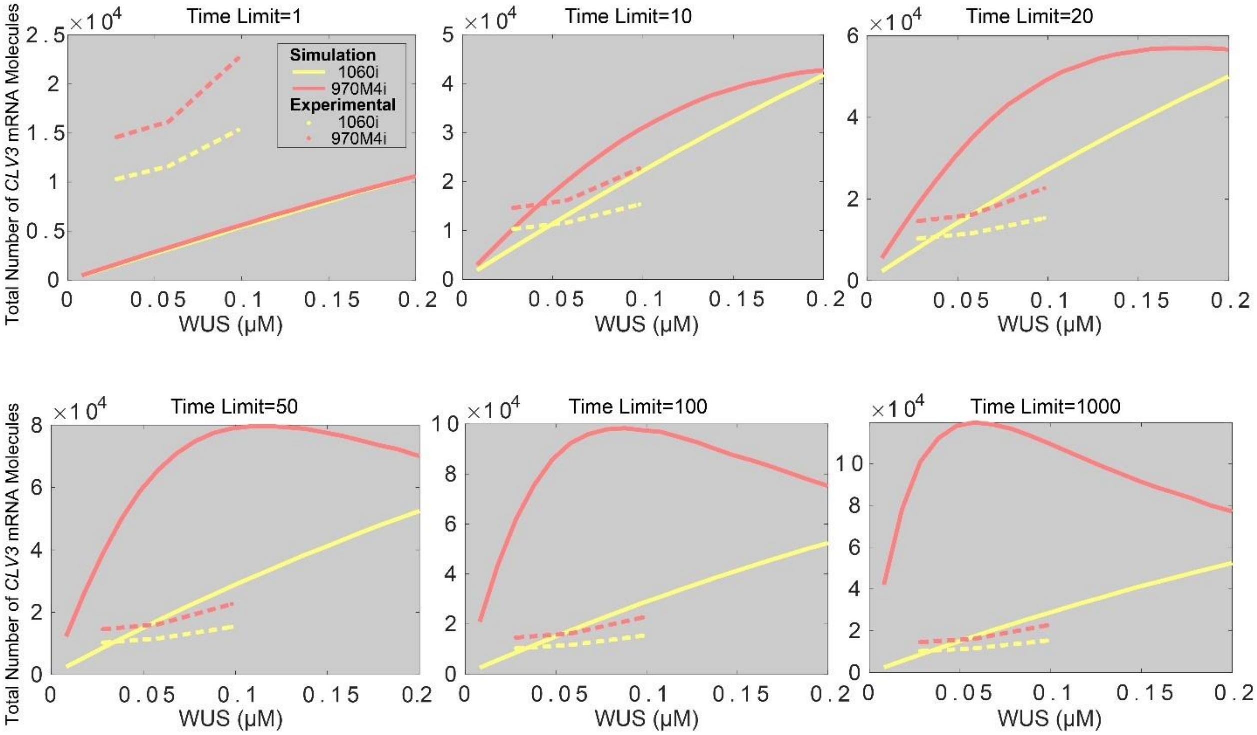

Fig. S5. The effect of WUS residence time limit on *CLV3* expression.

Scaled simulated values of *CLV3* mRNA production with increasing WUS concentrations is shown for the highest (970M4i [Solid red lines]) and the lowest (1060i [Solid yellow lines]) affinity cis-elements. The simulated data is compared to the *pCLV3::H2b-mYFP* levels in different cell layers shown as 3 dots (L1, L2 and L3) connected with dashed lines for 1060i (yellow) and 970M4i (red). Increasing the WUS residence time limit increases *CLV3* mRNA production and shifts the peak production to a lower WUS concentration range, especially in the case of higher affinity cis-element.

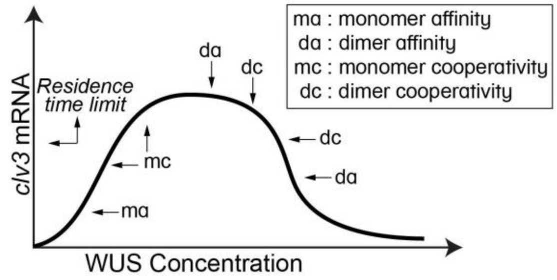

Fig. S6. The effect of RNA Pol II, WUS binding dynamics and WUS residence time limit on WUS concentration-dependent *CLV3* expression.

Increasing the affinity or cooperativity of WUS monomer leads to an increase in *CLV3* expression and shifts expression to a lower range of WUS concentration. Increasing the affinity or cooperativity of WUS dimer leads to *CLV3* repression, which decreases *CLV3* expression at high WUS concentration. Increasing the WUS residence time limit increases *CLV3* activation and shifts the activation to a lower range of WUS concentration.

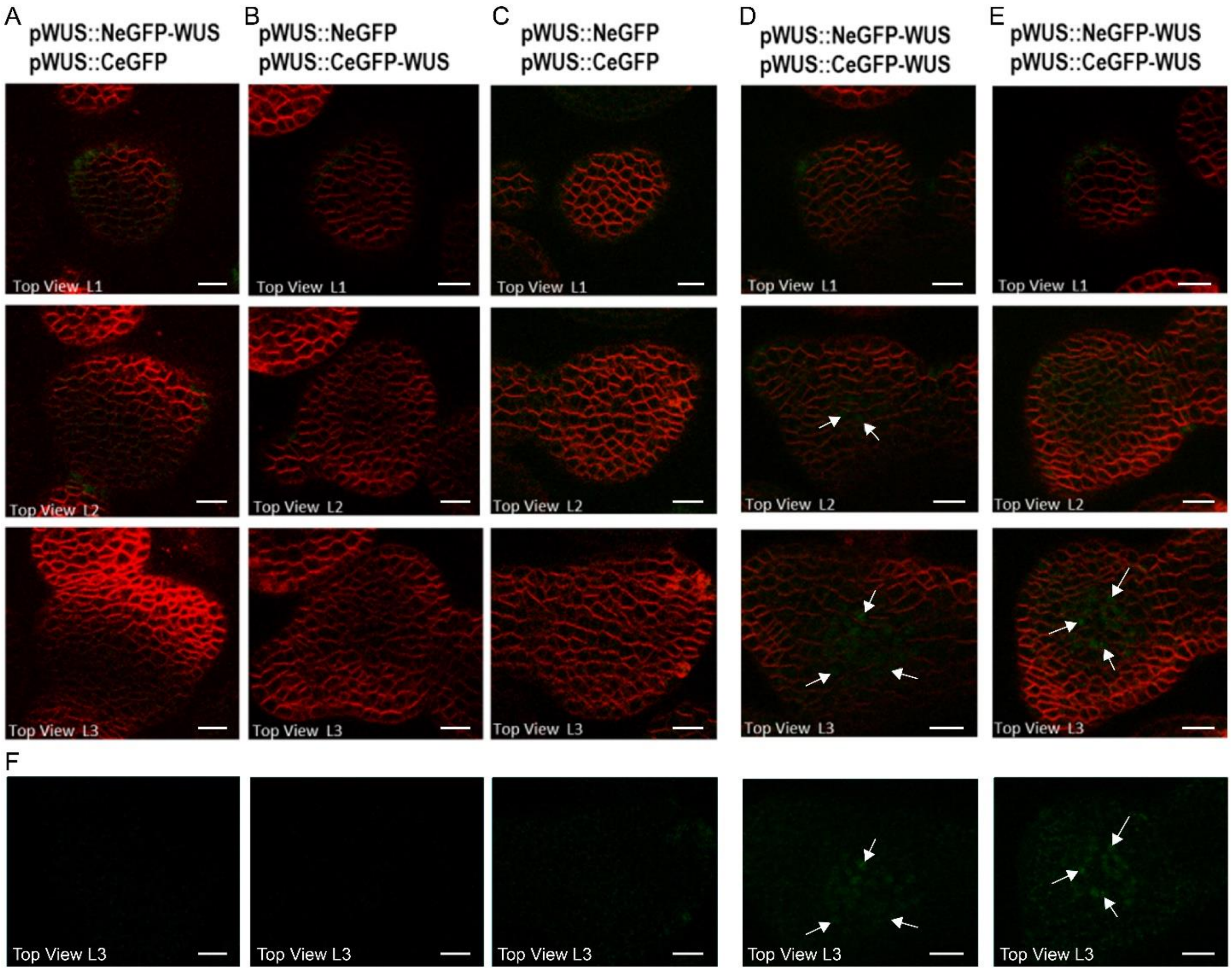

Fig. S7. *In planta* WUS protein homodimerization. The three rows represent the Z slices showing the Layer 1, Layer 2 and Layer 3 of SAM. (**A-E**) The WUS promoter was used to express all the BIFC construct in the wild-type background. (**A**) Plant expressing N' terminal fragment of eGFP translationally fused to WUS and C' terminal fragment of eGFP. (**B**) Plant expressing N' terminal fragment of eGFP and C' terminal fragment of eGFP translationally fused to WUS. (**C**) Plant expressing N' terminal fragment of eGFP and C' terminal fragment of eGFP. (**D** and **E**) Two independent lines expressing BiFC constructs of N' terminal fragment of eGFP translationally fused to WUS and C' terminal fragment of eGFP translationally fused to WUS. Arrows point to eGFP fluorescence (green) and FM4-64 staining is shown in red. (**F**) Images of Layer 3 shown in row 3, with the red channel turned off. Scale bar = 20 µm.

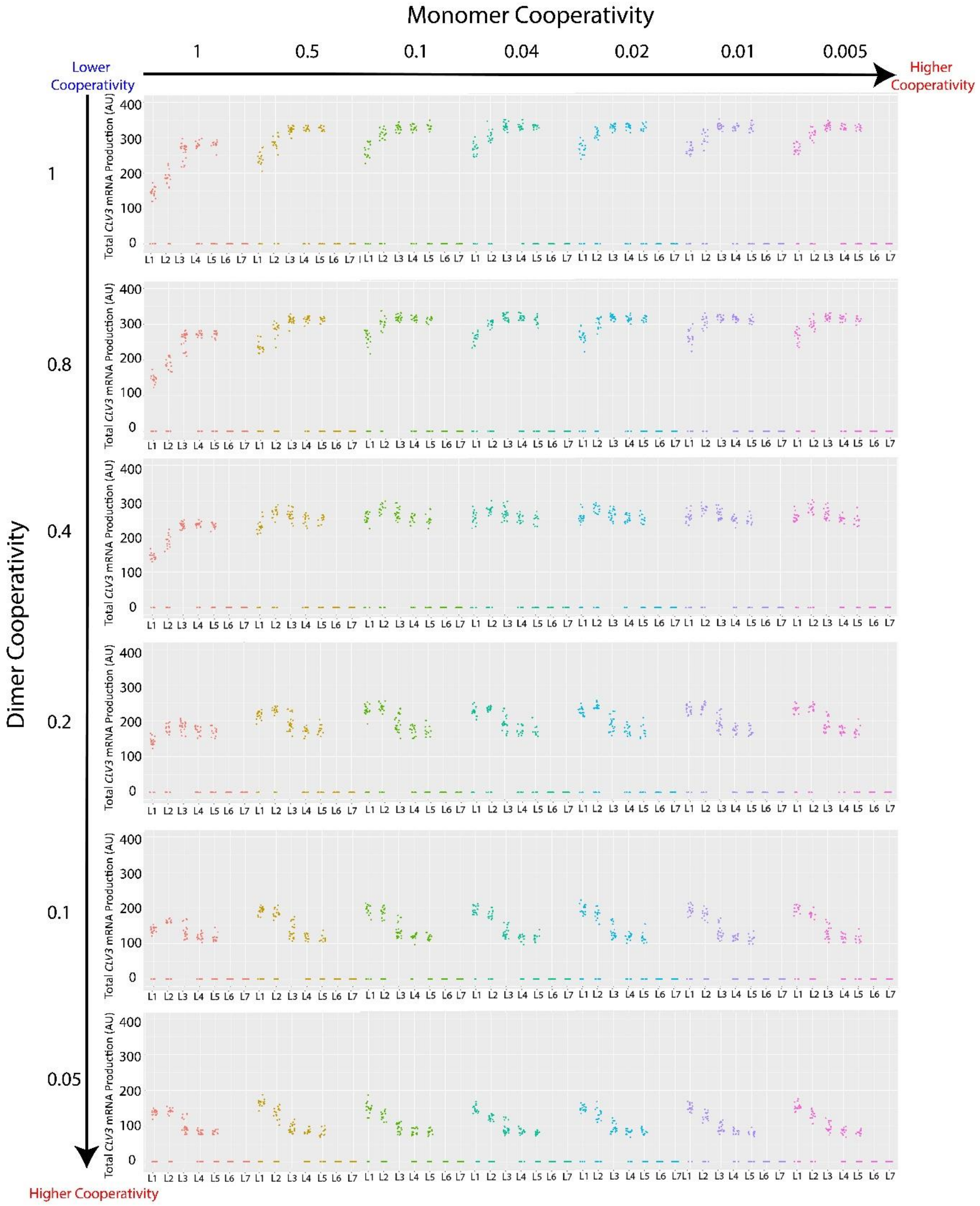

Fig. S8. Independent perturbations of the WUS monomer or dimer cooperativity.

The effect of changes in monomer cooperativity (from 1 to 0.005) and dimer cooperativity (from 1 to 0.05) on *CLV3* activation. The direction of the arrows indicate an increase in cooperativity. The result for each combination of monomer and dimer cooperativity value is represented by the graph at their intersection. The individual graphs represent the *CLV3* activation in different cell layers (L1 to L7) of simulated SAMs under the cooperativity levels noted for each simulation. The dots represent the values of the *CLV3* signal for individual simulated cells. AU, arbitrary unit.

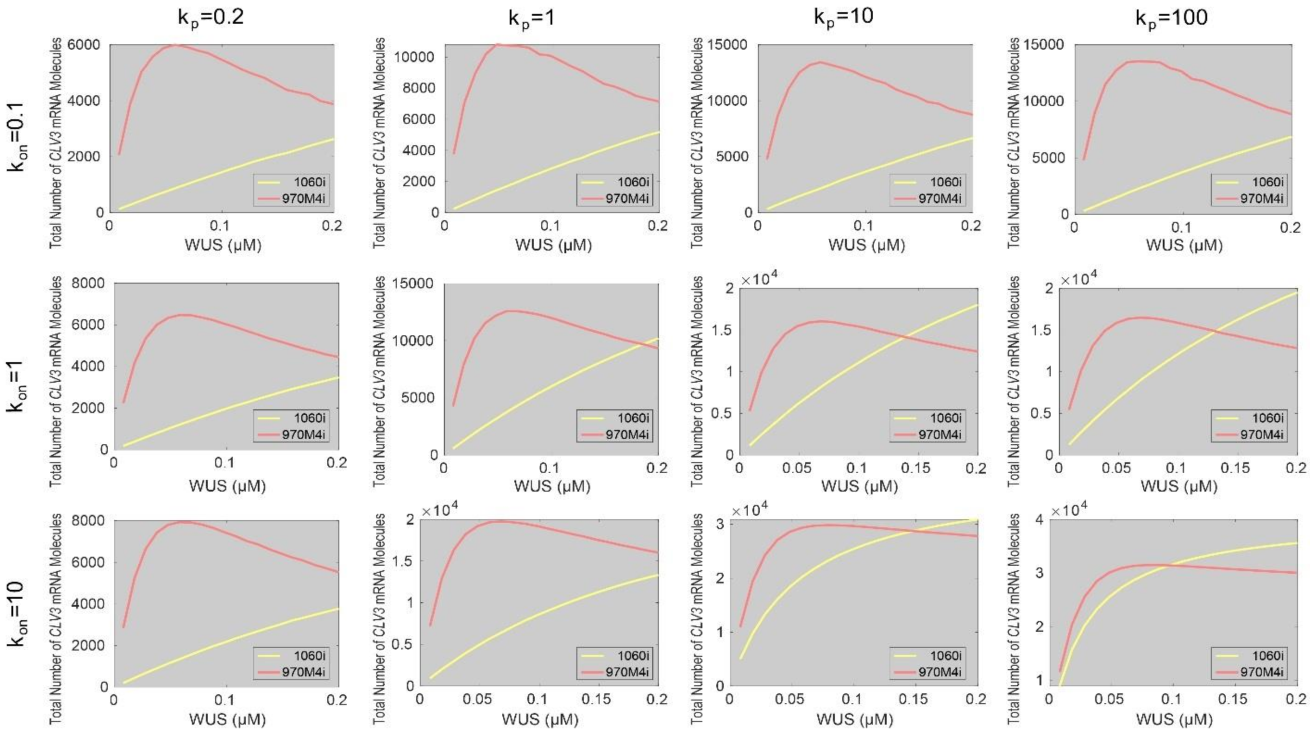

Fig. S9. Sensitivity analysis showing the effect of different k_{on}, k_p values on the behavior of highest and lowest affinity single cis-elements.

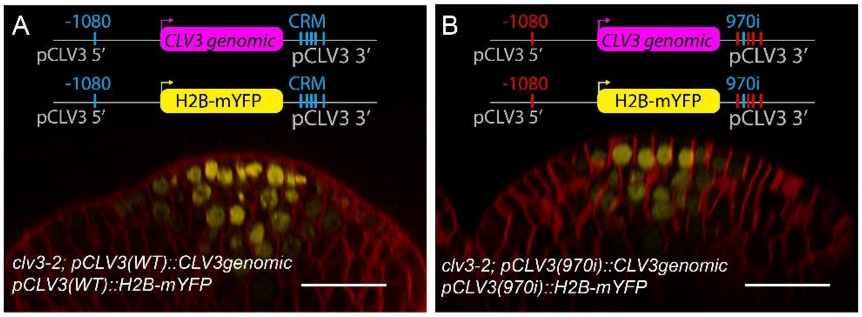

Fig. S10. Reactivation of mutant *CLV3* promoter (970i) in *clv3* complemented background. (**A**) *clv3-2* mutants complemented with the wild-type CLV3 promoter expressing *CLV3* genomic sequence [*pCLV3(WT)::CLV3* genomic]and showing the wild-type *CLV3* reporter [*pCLV3(WT)::H2B-mYFP*] expression. (**B**) *clv3-2* mutants complemented with the 970i *CLV3* promoter expressing *CLV3* genomic [*pCLV3(970i)::CLV3* genomic] sequence and showing the 970i *CLV3* reporter [*pCLV3(970i)::H2B-mYFP*]. Scale bar = 20 µm.

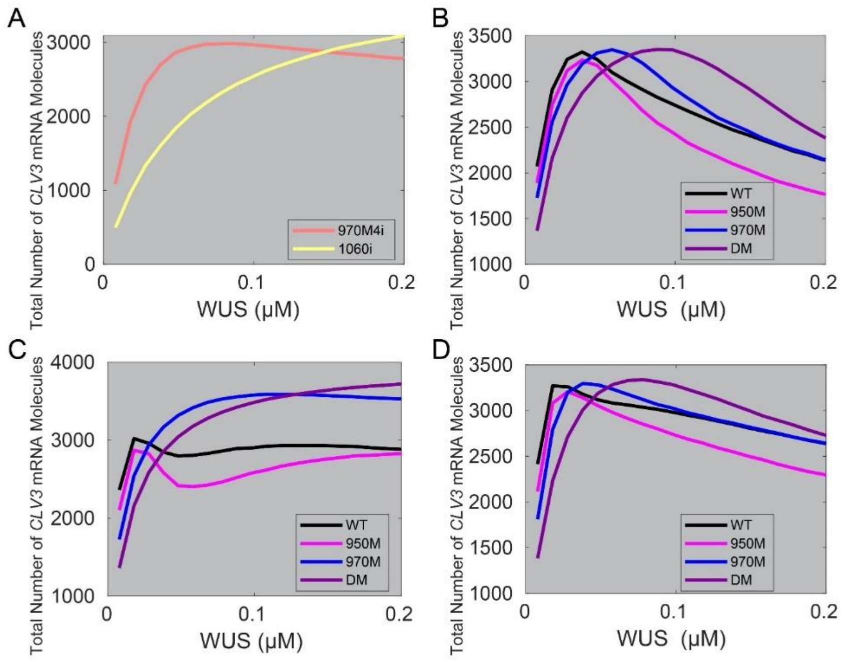

Fig. S11. Single cell simulation results of WUS mediated activation of *CLV3* by using fast dynamics (k_{on}=10 and k_p=10). (**A**) Single cis-element behavior for 970M4i and 1060i. (**B**) Multiple cis-element behaviors with low cooperativity between neighboring cis-elements only. (**C**) Multiple cis-element behaviors with distance dependent cooperativity. (**D**) Multiple cis-element behaviors with high cooperativity between neighboring cis-elements only.

Table S1. Experimentally derived KD values from EMSA.

K_d Values for the DNA binding domain of WUS to cis-elements probes were obtained from (*24*).

Parameter	Value	Definition	Unit
K_d	0.05830	Dissociation constant of 970M4i	μM
K_d	0.9571	Dissociation constant of 950i	μM
K_d	0.1855	Dissociation constant of 970i	μM
K_d	0.3663	Dissociation constant of 997i	μM
K_d	0.5652	Dissociation constant of 1007i	μM
K_d	1.249	Dissociation constant of 1060i	μM

Table S2. Parameter Values Used in Single Cell Model.

All values in this table were calibrated in this model.

Parameter	Value	Definition	Unit
k_p	0.2-100	Pol II binding rate	$\mu M^{-1}s^{-1}$
k_{on}	0.1-10	Monomer, dimer binding rate	$\mu M^{-1}s^{-1}$
t_{delay}	4	Time delay for the recruitment of successive Pol II	s
a_m	1.1	Parameter determining the level of monomer cooperativity	-
b_m	50	Parameter determining the distance threshold of monomer cooperativity	$2bp$
a_d	1.1	Parameter determining the level of dimer cooperativity	-
b_d	50	Parameter determining the distance threshold of dimer cooperativity	$2bp$
RTL	10	Residence time limit	s
t_{final}	1.6×10^6	Final time of a simulation	s
a_c	0.3	Parameter determining the nonlinearity of the distance function for the cooperativity	-

Table S3. *CLV3* mRNA Production Parameters used in the 3D cell-based model.

Parameter	Value	Definition	Unit	Reference
clv3CLV3P	3.0	Rate of *CLV3* mRNA production	s^{-1}	Calibrated in model
clv3SourceWidth	3	Radius of *CLV3* activation domain in x-y plane	a.u.	Experimentally determined
clv3WusSatPoint	425	Maximum effective WUS concentration	-	Calibrated in model
clv3CooptMonEffect	0.01	Parameter determining the level of monomer cooperativity	-	Calibrated in model
clv3CooptDimEffect	0.20	Parameter determining the level of dimer cooperativity	-	Calibrated in model
polTimeLimit	5	Time delay for Pol II firing	s	Experimentally determined
n_{970}	3	The minimal exponent in the cooperativity associated with 970	-	Calibrated in model
k_p	0.1	Pol II binding parameter (*clv3polBaseBindAffinity*)	s^{-1}	Calibrated in model

Table S4. Primers used in this study.

Construct	Primer Name	Sequence
**CLAVATA3 mutant promoter**		
pCLV3 950M	Fwd	CGTACCCCCAAATTTTCCCAACGGTACATTGC
pCLV3 950M	Rev	TTTTCAATTGTCAATGCAAATACCCCATGG
pCLV3 970M	Fwd	GGTATTTGCATTGACAATTGAAAACGTAC
pCLV3 970M	Rev	CCATGGATGTGATAGTCACAATTAAAC
pCLV3 997M	Fwd	GTGACTATCACATCCATTAATTATTTGC
pCLV3 997M	Rev	AATGGAACATACAATAATAAAAATGATGATG
pCLV3 1007M	Fwd	GATTCGATGATGTGGTGGGAAGG
pCLV3 1007M	Rev	ATCATCATCATTTTTGGGGTTGTATGTT
pCLV3 1060M	Fwd	GTCGGTTCCCCTTATCCTTCCCACCACATCATC
pCLV3 1060M	Rev	TTTGGGGCAGTGACAGGCAGTGTCAGTG
**Double Space Around 997 primers**		
pCLV3 DS-C	Rev	TTATTATTGTATGTTTTGTATGTTTAATTGTGACTATCAC ATCCTGTGACTATCACATCC
pCLV3 RB	Fwd	ATAAAAAAATGATGATGATGATTCGATGATGTGGTGGG AAG
**EMSA probes**		
**Cis-element**	**Orientation**	**Sequence**
970	Fwd	CAATTGTCAATGCAAATAATTAATGGATGTG
970	Rev	CACATCCATTAATTATTTGCATTGACAATTG
997	Fwd	TTATTGTATGTTTAATTGTGACTAT
997	Rev	ATAGTCACAATTAAACATACAATAA
970+997	Fwd	CAATTGTCAATGCAAATAATTAATGGATGTGATAGTCAC AATTAAACATACAATA
970+997	Rev	TATTGTATGTTTAATTGTGACTATCACATCCATTAATTAT TTGCATTGACAATTG

970m+997	Fwd	CAATTGTCAATGCAAAGGGGGGGGGATGTGATAGTCA CAATTAAACATACAATA
970m+997	Rev	TATTGTATGTTTAATTGTGACTATCACATCCCCCCCCCT TTGCATTGACAATTG
970+997m	Fwd	CAATTGTCAATGCAAATAATTAATGGATGTGATAGTCAC AGGGGAACATACAATA
970+997m	Rev	TATTGTATGTTCCCCTGTGACTATCACATCCATTAATTAT TTGCATTGACAATTG
970M4+997	Fwd	CAATTGTCAATGCAAATAACTAATGGATGTGATAGTCAC AATTAAACATACAATA
970M4+997	Rev	TATTGTATGTTTAATTGTGACTATCACATCCATTAGTTAT TTGCATTGACAATTG
970M4+997m	Fwd	CAATTGTCAATGCAAATAACTAATGGATGTGATAGTCAC AGGGGAACATACAATA
970M4+997m	Rev	TATTGTATGTTCCCCTGTGACTATCACATCCATTAGTTAT TTGCATTGACAATTG
Double Space 970+997	Fwd	CAATTGTCAATGCAAATAATTAATGGATGTGATAGTCAC AGGATGTGATAGTCACAATTAAACATACAATA
Double Space 970+997	Rev	TATTGTATGTTTAATTGTGACTATCACATCCTGTGACTAT CACATCCATTAATTATTTGCATTGACAATTG
	**BiFC Cloning**	
ΔCeGFP	NeGFP -Fw	GGATCCATGGAGCCGCCACAGCATCAG
	NeGFP-Rev	GGCCATGATATAGACGTTGTGGCTGTTG
ΔNeGFP	CeGFP-Fw	GACAAGCAGAAGAACGGCATCAAGGTG
	CeGFP-Rev	CATGGCGCGCCATGGTGAAGGAGCCCTG
ΔWUS	ΔWUS-Fw	TGAACTAGGCCTGCAAGGGCG
	ΔWUS-Rev	GGATCCCTTGTACAGCTCGTC

Table S5. Cis-element mutant sequence library.

CLV3 Promoter name	Sequence
WT	5’tttATTAgtacgttttcaattgtcaatgcaaaTAATTAATggatgtgatagtcacaATTAaa<br>catacaaTAATaaaaatgatgatgatgattcgatgatgtggtgggaaggataaATTAaa-3’
950M	5’tttGGGGgtacgttttcaattgtcaatgcaaaTAATTAATggatgtgatagtcacaATTAaa<br>catacaaTAATaaaaatgatgatgatgattcgatgatgtggtgggaaggataaATTAaa-3’
970M	5’tttATTAgtacgttttcaattgtcaatgcaaaTACCCCATggatgtgatagtcacaATTAaa<br>catacaaTAATaaaaatgatgatgatgattcgatgatgtggtgggaaggataaATTAaa-3’
997M	5’tttATTAgtacgttttcaattgtcaatgcaaaTAATTAATggatgtgatagtcacaATGGaa<br>catacaaTAATaaaaatgatgatgatgattcgatgatgtggtgggaaggataaATTAaa-3’
1007M	5’tttATTAgtacgttttcaattgtcaatgcaaaTAATTAATggatgtgatagtcacaATTAaa<br>catacaaCCCCaaaaatgatgatgatgattcgatgatgtggtgggaaggataaATTAaa-3’
1060M	5’tttATTAgtacgttttcaattgtcaatgcaaaTAATTAATggatgtgatagtcacaATTAaa<br>catacaaTAATaaaaatgatgatgatgattcgatgatgtggtgggaaggataaGGGGaa-3’
970M4	5’tttATTAgtacgttttcaattgtcaatgcaaaTAACTAATggatgtgatagtcacaATTAaa<br>catacaaTAATaaaaatgatgatgatgattcgatgatgtggtgggaaggataaATTAaa-3’
950i	5’tttATTAgtacgttttcaattgtcaatgcaaaTACCCCATggatgtgatagtcacaATGGaa<br>catacaaCCCCaaaaatgatgatgatgattcgatgatgtggtgggaaggataaGGGGaa-3’
970i	5’tttGGGGgtacgttttcaattgtcaatgcaaaTAATTAATggatgtgatagtcacaATGGaa<br>catacaaCCCCaaaaatgatgatgatgattcgatgatgtggtgggaaggataaGGGGaa-3’
997i	5’tttGGGGgtacgttttcaattgtcaatgcaaaTACCCCATggatgtgatagtcacaATTAaa<br>catacaaCCCCaaaaatgatgatgatgattcgatgatgtggtgggaaggataaGGGGaa-3’
1007i	5’tttGGGGgtacgttttcaattgtcaatgcaaaTACCCCATggatgtgatagtcacaATGGaa<br>catacaaTAATaaaaatgatgatgatgattcgatgatgtggtgggaaggataaGGGGaa-3’
1060i	5’tttGGGGgtacgttttcaattgtcaatgcaaaTACCCCATggatgtgatagtcacaATGGaa<br>catacaaCCCCaaaaatgatgatgatgattcgatgatgtggtgggaaggataaATTAaa-3’
970M4i	5’tttGGGGgtacgttttcaattgtcaatgcaaaTAACTAATggatgtgatagtcacaATGGaa<br>catacaaCCCCaaaaatgatgatgatgattcgatgatgtggtgggaaggataaGGGGaa-3’

Table S6. Predicted TAAT clusters in promoter of WUS upregulated target genes.
Summary statistics of predicted TAAT clusters in the promoters of WUS upregulated genes.

Table S7. Predicted TAAT clusters in promoters of WUS downregulated target genes.
Summary statistics of predicted TAAT clusters in the promoters of WUS downregulated genes.

Data S1. (separate file)

Individual data points, means, N and P values are arranged by figure and panel.

REFERENCES AND NOTES

1. M. Levine, Transcriptional enhancers in animal development and evolution. *Curr. Biol.* **20**, R754–R763 (2010).

2. C.-T. Ong, V. G. Corces, Enhancer function: New insights into the regulation of tissue-specific gene expression. *Nat. Rev. Genet.* **12**, 283–293 (2011).

3. F. Spitz, E. E. M. Furlong, Transcription factors: From enhancer binding to developmental control. *Nat. Rev. Genet.* **13**, 613–626 (2012).

4. J. Banerji, S. Rusconi, W. Schaffner, Expression of a beta-globin gene is enhanced by remote SV40 DNA sequences. *Cell* **27**, 299–308 (1981).

5. M. Slattery, T. Riley, P. Liu, N. Abe, P. Gomez-Alcala, I. Dror, T. Zhou, R. Rohs, B. Honig, H. J. Bussemaker, R. S. Mann, Cofactor binding evokes latent differences in DNA binding specificity between Hox proteins. *Cell* **147**, 1270–1282 (2011).

6. G. Struhl, K. Struhl, P. M. Macdonald, The gradient morphogen *bicoid* is a concentration-dependent transcriptional activator. *Cell* **57**, 1259–1273 (1989).

7. W. D. Fakhouri, A. Ay, R. Sayal, J. Dresch, E. Dayringer, D. N. Arnosti, Deciphering a transcriptional regulatory code: Modeling short-range repression in the Drosophila embryo. *Mol. Syst. Biol.* **6**, 341 (2010).

8. R. Joshi, J. M. Passner, R. Rohs, R. Jain, A. Sosinsky, M. A. Crickmore, V. Jacob, A. K. Aggarwal, B. Honig, R. S. Mann, Functional specificity of a Hox protein mediated by the recognition of minor groove structure. *Cell* **131**, 530–543 (2007).

9. J. Crocker, N. Abe, L. Rinaldi, A. P. McGregor, N. Frankel, S. Wang, A. Alsawadi, P. Valenti, S. Plaza, F. Payre, R. S. Mann, D. L. Stern, Low affinity binding site clusters confer Hox specificity and regulatory robustness. *Cell* **160**, 191–203 (2015).

10. B. P. Berman, Y. Nibu, B. D. Pfeiffer, P. Tomancak, S. E. Celniker, M. Levine, G. M. Rubin, M. B. Eisen, Exploiting transcription factor binding site clustering to identify cis-regulatory modules involved in pattern formation in the Drosophila genome. *Proc. Natl. Acad. Sci. U.S.A.* **99**, 757–762 (2002).

11. A. P. Lifanov, V. J. Makeev, A. G. Nazina, D. A. Papatsenko, Homotypic regulatory clusters in Drosophila. *Genome Res.* **13**, 579–588 (2003).

12. S. Small, A. Blair, M. Levine, Regulation of even-skipped stripe 2 in the Drosophila embryo. *EMBO J.* **11**, 4047–4057 (1992).

13. Y. T. Ip, R. E. Park, D. Kosman, E. Bier, M. Levine, The dorsal gradient morphogen regulates stripes of rhomboid expression in the presumptive neuroectoderm of the Drosophila embryo. *Genes Dev.* **6**, 1728–1739 (1992).

14. W. Driever, C. Nüsslein-Volhard, The bicoid protein is a positive regulator of *hunchback* transcription in the early *Drosophila* embryo. *Nature* **337**, 138–143 (1989).

15. J. Gaudet, S. E. Mango, Regulation of organogenesis by the *Caenorhabditis elegans* FoxA protein PHA-4. *Science* **295**, 821–825 (2002).

16. J. Jiang, M. Levine, Binding affinities and cooperative interactions with bHLH activators delimit threshold responses to the dorsal gradient morphogen. *Cell* **72**, 741–752 (1993).

17. S. Rowan, T. Siggers, S. A. Lachke, Y. Yue, M. L. Bulyk, R. L. Maas, Precise temporal control of the eye regulatory gene Pax6 via enhancer-binding site affinity. *Genes Dev.* **24**, 980–985 (2010).

18. L. Wolpert, Positional information and the spatial pattern of cellular differentiation. *J. Theor. Biol.* **25**, 1–47 (1969).

19. J. Jiang, D. Kosman, Y. T. Ip, M. Levine, The dorsal morphogen gradient regulates the mesoderm determinant twist in early Drosophila embryos. *Genes Dev.* **5**, 1881–1891 (1991).

20. D. S. Parker, M. A. White, A. I. Ramos, B. A. Cohen, S. Barolo, The cis-regulatory logic of Hedgehog gradient responses: Key roles for gli binding affinity, competition, and cooperativity. *Sci. Signal.* **4**, ra38 (2011).

21. K. F. Mayer, H. Schoof, A. Haecker, M. Lenhard, G. Jürgens, T. Laux, Role of WUSCHEL in regulating stem cell fate in the Arabidopsis shoot meristem. *Cell* **95**, 805–815 (1998).

22. H. Schoof, M. Lenhard, A. Haecker, K. F. X. Mayer, G. Jürgens, T. Laux, The stem cell population of Arabidopsis shoot meristems is maintained by a regulatory loop between the CLAVATA and WUSCHEL genes. *Cell* **100**, 635–644 (2000).

23. R. K. Yadav, M. Perales, J. Gruel, T. Girke, H. Jönsson, G. V. Reddy, WUSCHEL protein movement mediates stem cell homeostasis in the Arabidopsis shoot apex. *Genes Dev.* **25**, 2025–2030 (2011).

24. M. Perales, K. Rodriguez, S. Snipes, R. K. Yadav, M. Diaz-Mendoza, G. V. Reddy, Threshold-dependent transcriptional discrimination underlies stem cell homeostasis. *Proc. Natl. Acad. Sci. U.S.A.* **113**, E6298–E6306 (2016).

25. S. E. Clark, R. W. Williams, E. M. Meyerowitz, The CLAVATA1 gene encodes a putative receptor kinase that controls shoot and floral meristem size in Arabidopsis. *Cell* **89**, 575–585 (1997).

26. U. Brand, J. C. Fletcher, M. Hobe, E. M. Meyerowitz, R. Simon, Dependence of stem cell fate in Arabidopsis on a feedback loop regulated by CLV3 activity. *Science* **289**, 617–619 (2000).

27. R. K. Yadav, M. Perales, J. Gruel, C. Ohno, M. Heisler, T. Girke, H. Jönsson, G. V. Reddy, Plant stem cell maintenance involves direct transcriptional repression of differentiation program. *Mol. Syst. Biol.* **9**, 654 (2013).

28. C. Koppermann, "Crystal structure of the WUSCHEL homeodomain," thesis, Universität zu Köln, Cologne, Germany (2017).

29. J. Sloan, J. P. Hakenjos, M. Gebert, O. Ermakova, A. Gumiero, G. Stier, K. Wild, I. Sinning, J. U. Lohmann, Structural basis for the complex DNA binding behavior of the plant stem cell regulator WUSCHEL. *Nat. Commun.* **11**, 2223 (2020).

30. J. Reinitz, S. Hou, D. H. Sharp, Transcriptional control in *Drosophila*. *Complexus* **1**, 54–64 (2003).

31. M. A. Shea, G. K. Ackers, The OR control system of bacteriophage lambda. A physical-chemical model for gene regulation. *J. Mol. Biol.* **181**, 211–230 (1985).

32. X. He, M. A. H. Samee, C. Blatti, S. Sinha, Thermodynamics-based models of transcriptional regulation by enhancers: The roles of synergistic activation, cooperative binding and short-range repression. *PLOS Comput. Biol.* **6**, e1000935 (2010).

33. D. Chu, N. R. Zabet, B. Mitavskiy, Models of transcription factor binding: Sensitivity of activation functions to model assumptions. *J. Theor. Biol.* **257**, 419–429 (2009).

34. M. S. Sherman, B. A. Cohen, Thermodynamic state ensemble models of cis-regulation. *PLOS Comput. Biol.* **8**, e1002407 (2012).

35. D. T. Gillespie, A general method for numerically simulating the stochastic time evolution of coupled chemical reactions. *J. Comput. Phys.* **22**, 403–434 (1976).

36. J. Swift, G. M. Coruzzi, A matter of time—How transient transcription factor interactions create dynamic gene regulatory networks. *Biochim. Biophys. Acta Gene Regul. Mech.* **1860**, 75–83 (2017).

37. T. O'Brien, J. T. Lis, Rapid changes in Drosophila transcription after an instantaneous heat shock. *Mol. Cell. Biol.* **13**, 3456–3463 (1993).

38. G. K. Ackers, A. D. Johnson, M. A. Shea, Quantitative model for gene regulation by lambda phage repressor. *Proc. Natl. Acad. Sci. U.S.A.* **79**, 1129–1133 (1982).

39. H. G. Garcia, R. Phillips, Quantitative dissection of the simple repression input-output function. *Proc. Natl. Acad. Sci. U.S.A.* **108**, 12173–12178 (2011).

40. J. Gertz, E. D. Siggia, B. A. Cohen, Analysis of combinatorial *cis*-regulation in synthetic and genomic promoters. *Nature* **457**, 215–218 (2009).

41. E. Segal, T. Raveh-Sadka, M. Schroeder, U. Unnerstall, U. Gaul, Predicting expression patterns from regulatory sequence in *Drosophila* segmentation. *Nature* **451**, 535–540 (2008).

42. R. P. Zinzen, C. Girardot, J. Gagneur, M. Braun, E. E. M. Furlong, Combinatorial binding predicts spatio-temporal *cis*-regulatory activity. *Nature* **462**, 65–70 (2009).

43. R. Amit, H. G. Garcia, R. Phillips, S. E. Fraser, Building enhancers from the ground up: A synthetic biology approach. *Cell* **146**, 105–118 (2011).

44. E. Davidson, *The Regulatory Genome* (Academic Press, 2006).

45. H. G. Garcia, A. Sanchez, J. Q. Boedicker, M. Osborne, J. Gelles, J. Kondev, R. Phillips, Operator sequence alters gene expression independently of transcription factor occupancy in bacteria. *Cell Rep.* **2**, 150–161 (2012).

46. C. R. Lickwar, F. Mueller, S. E. Hanlon, J. G. McNally, J. D. Lieb, Genome-wide protein–DNA binding dynamics suggest a molecular clutch for transcription factor function. *Nature* **484**, 251–255 (2012).

47. J. R. Lipford, G. T. Smith, Y. Chi, R. J. Deshaies, A putative stimulatory role for activator turnover in gene expression. *Nature* **438**, 113–116 (2005).

48. S. H. Spoel, Z. Mou, Y. Tada, N. W. Spivey, P. Genschik, X. Dong, Proteasome-mediated turnover of the transcription coactivator NPR1 plays dual roles in regulating plant immunity. *Cell* **137**, 860–872 (2009).

49. F. Geng, S. Wenzel, W. P. Tansey, Ubiquitin and proteasomes in transcription. *Annu. Rev. Biochem.* **81**, 177–201 (2012).

50. A. Sundqvist, J. Ericsson, Transcription-dependent degradation controls the stability of the SREBP family of transcription factors. *Proc. Natl. Acad. Sci. U.S.A.* **100**, 13833–13838 (2003).

51. A. Plong, K. Rodriguez, M. Alber, W. Chen, G. V. Reddy, CLAVATA3 mediated simultaneous control of transcriptional and post-translational processes provides robustness to the WUSCHEL gradient. *Nat. Commun.* **12**, 6361 (2021).

52. S. A. Snipes, K. Rodriguez, A. E. DeVries, K. N. Miyawaki, M. Perales, M. Xie, G. V. Reddy, Cytokinin stabilizes WUSCHEL by acting on the protein domains required for nuclear enrichment and transcription. *PLOS Genet.* **14**, e1007351 (2018).

3. K. Rodriguez, M. Perales, S. Snipes, R. K. Yadav, M. Diaz-Mendoza, G. V. Reddy, DNA-dependent homodimerization, sub-cellular partitioning, and protein destabilization control WUSCHEL levels and spatial patterning. *Proc. Natl. Acad. Sci. U.S.A.* **113**, E6307–E6315 (2016).

54. R. Sayal, J. M. Dresch, I. Pushel, B. R. Taylor, D. N. Arnosti, Quantitative perturbation-based analysis of gene expression predicts enhancer activity in early Drosophila embryo. *Elife* **5**, e08445 (2016).

55. Y. Zhou, X. Liu, E. M. Engstrom, Z. L. Nimchuk, J. L. Pruneda-Paz, P. T. Tarr, A. Yan, S. A. Kay, E. M. Meyerowitz, Control of plant stem cell function by conserved interacting transcriptional regulators. *Nature* **517**, 377–380 (2015).

56. Y. H. Su, C. Zhou, Y. J. Li, Y. Yu, L. P. Tang, W. J. Zhang, W. J. Yao, R. Huang, T. Laux, X. S. Zhang, Integration of pluripotency pathways regulates stem cell maintenance in the *Arabidopsis* shoot meristem. *Proc. Natl. Acad. Sci. U.S.A.* **117**, 22561–22571 (2020).

57. M. Kieffer, Y. Stern, H. Cook, E. Clerici, C. Maulbetsch, T. Laux, B. Davies, Analysis of the transcription factor WUSCHEL and its functional homologue in Antirrhinum reveals a potential mechanism for their roles in meristem maintenance. *Plant Cell* **18**, 560–573 (2006).

58. B. Senay-Aras, W. Chen. Stochastic cis-elements binding model (2022); https://zenodo.org/badge/latestdoi/349283086.

59. A. Do, MeristemBasic_p (2022); https://zenodo.org/badge/latestdoi/501822165.

60. A. Do, BasicCisElementAnalyzer (2022); https://zenodo.org/badge/latestdoi/498280124.

61. M. R. Roussel, R. Zhu, Validation of an algorithm for delay stochastic simulation of transcription and translation in prokaryotic gene expression. *Phys. Biol.* **3**, 274–284 (2006).

62. E. Azpeitia, A. Wagner, Short residence times of DNA-bound transcription factors can reduce gene expression noise and increase the transmission of information in a gene regulation system. *Front. Mol. Biosci.* **7**, 67 (2020).

63. P. S. Gutierrez, D. Monteoliva, L. Diambra, Cooperative binding of transcription factors promotes bimodal gene expression response. *PLOS ONE* **7**, e44812 (2012).

64. U. Brand, M. Grünewald, M. Hobe, R. Simon, Regulation of CLV3 expression by two homeobox genes in Arabidopsis. *Plant Physiol.* **129**, 565–575 (2002).
